# A noncanonical function of EIF4E limits ALDH1B1 activity and increases susceptibility to ferroptosis

**DOI:** 10.1038/s41467-022-34096-w

**Published:** 2022-10-23

**Authors:** Xin Chen, Jun Huang, Chunhua Yu, Jiao Liu, Wanli Gao, Jingbo Li, Xinxin Song, Zhuan Zhou, Changfeng Li, Yangchun Xie, Guido Kroemer, Jinbao Liu, Daolin Tang, Rui Kang

**Affiliations:** 1 DAMP Laboratory, The Third Affiliated Hospital, Guangzhou Medical University, Guangzhou, China; 2 Guangzhou Municipal and Guangdong Provincial Key Laboratory of Protein Modification and Degradation, School of Basic Medical Sciences, Guangzhou Medical University, Guangzhou, China; 3 Affiliated Cancer Hospital & Institute of Guangzhou Medical University, Guangzhou, China; 4 Department of Surgery, UT Southwestern Medical Center, Dallas, TX USA; 5 Department of Orthopaedics, The Second Xiangya Hospital, Central South University, Changsha, China; 6 Department of Endoscopy Center, China-Japan Union Hospital of Jilin University, Changchun, Jilin 130033 China; 7 Department of Oncology, The Second Xiangya Hospital, Central South University, Changsha, Hunan China; 8 Centre de Recherche des Cordeliers, Equipe labellisée par la Ligue contre le cancer, Université de Paris Cité, Sorbonne Université, Inserm U1138, Institut Universitaire de France, Paris, France; 9 Metabolomics and Cell Biology Platforms, Gustave Roussy Cancer Campus, 94800 Villejuif, France; 10 Institut du Cancer Paris CARPEM, Department of Biology, Hôpital Européen Georges Pompidou, AP-HP, 75015 Paris, France

**Keywords:** Cell death, Gastrointestinal cancer, Mechanisms of disease

## Abstract

Ferroptosis is a type of lipid peroxidation-dependent cell death that is emerging as a therapeutic target for cancer. However, the mechanisms of ferroptosis during the generation and detoxification of lipid peroxidation products remain rather poorly defined. Here, we report an unexpected role for the eukaryotic translation initiation factor EIF4E as a determinant of ferroptotic sensitivity by controlling lipid peroxidation. A drug screening identified 4EGI-1 and 4E1RCat (previously known as EIF4E-EIF4G1 interaction inhibitors) as powerful inhibitors of ferroptosis. Genetic and functional studies showed that EIF4E (but not EIF4G1) promotes ferroptosis in a translation-independent manner. Using mass spectrometry and subsequent protein-protein interaction analysis, we identified EIF4E as an endogenous repressor of ALDH1B1 in mitochondria. ALDH1B1 belongs to the family of aldehyde dehydrogenases and may metabolize the aldehyde substrate 4-hydroxynonenal (4HNE) at high concentrations. Supraphysiological levels of 4HNE triggered ferroptosis, while low concentrations of 4HNE increased the cell susceptibility to classical ferroptosis inducers by activating the NOX1 pathway. Accordingly, EIF4E-dependent ALDH1B1 inhibition enhanced the anticancer activity of ferroptosis inducers in vitro and in vivo. Our results support a key function of EIF4E in orchestrating lipid peroxidation to ignite ferroptosis.

## Abstract (web summary)

Ferroptosis is lipid peroxidation-dependent cell death that has potential to be harnessed as a cancer therapeutic. Here, the authors show that the translation initiation factor eIF4E can repress ALDH1B1 independent of translation, increasing lipid peroxidation levels to promote ferroptosis.

## Introduction

Activating mutations in the RAS oncogene are the most common genetic abnormalities in human cancers. Researchers have sought effective RAS inhibitors for more than three decades. Historically, ferroptosis was discovered (and named) by screening small molecule compounds to selectively kill cancer cells with RAS mutations in an iron-dependent fashion^1^. Nowadays, it is known that ferroptosis occurs in both normal and tumor cells in a RAS-dependent or -independent manner^2^. Unlike caspase-dependent apoptosis and mixed-lineage kinase domain-like pseudokinase (MLKL)-mediated necroptosis, ferroptosis is mainly driven by unrestricted lipid peroxidation, a dynamic process initiated by an attack of reactive oxygen species (ROS) on lipids, especially polyunsaturated fatty acids (PUFAs) in cellular or organellar membranes^3^. This process is positively regulated by pro-oxidative enzymes, such as lipoxygenases (ALOXs)^4–7^, cytochrome P450 oxidoreductase (POR)^8,9^, and NADPH oxidases (NOXs)^1,10,11^. In contrast, multiple antioxidant systems, especially glutathione peroxidase 4 (GPX4) activation^12^, apoptosis-inducing factor mitochondria-associated 2 (AIFM2/FSP1)-dependent ubiquinol recycling^13,14^, and GTP cyclohydrolase 1 (GCH1)-mediated tetrahydrobiopterin production^15,16^, play a context-dependent role in preventing lipid peroxidation during ferroptosis. Although great progress has been made in understanding these ferroptosis-relevant regulators of lipid peroxidation, one key unresolved question is how lipid oxidation products trigger ferroptotic cell death^17,18^.

Translation initiation is the rate-limiting step of protein synthesis, and this process is fine-tuned by a family of proteins called eukaryotic initiation factors (EIFs). Specifically, eukaryotic initiation factor 4E (EIF4E) forms a translation initiation complex with eukaryotic initiation factor 4 gamma 1 (EIF4G1, also known as EIF4G), leading to the recruitment of mRNA to ribosomes^19^. Activated EIF4E is recognized as a potent oncoprotein due to its ability to increase protein synthesis, favoring cell proliferation and drug resistance^20,21^. EIF4E is widely overexpressed in human cancers as an important therapeutic target^22,23^. Therefore, although the benefit of the clinical response is uncertain, inhibiting the assembly of the EIF4E-EIF4G1 complex may constitute a reasonable antitumor strategy.

In this study, we provide evidence that, in cancer cells, EIF4E has an unexpected nontranslational function, rendering malignant cells particularly vulnerable to ferroptosis. Mechanistically, EIF4E physically interacts with aldehyde dehydrogenase 1 family member B1 (ALDH1B1) in membranes (especially mitochondria-associated membranes) to limit the ALDH1B1-mediated clearance of 4-hydroxynonenal (4HNE), which is not only a product of lipid peroxidation, but also a mediator of ferroptosis. These studies may provide a metabolic framework to understand the effector mechanism of ferroptosis.

## Results

### 4EGI-1 and 4E1RCat act as ferroptosis inhibitors

**Fig. 1:**
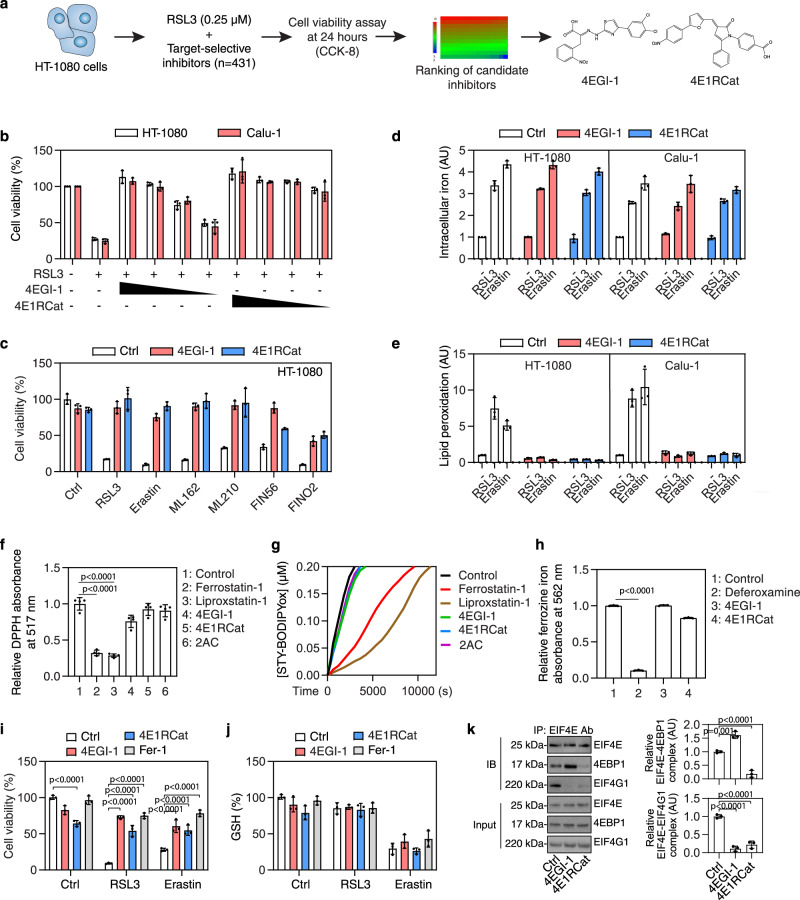
4EGI-1 and 4E1RCat act as ferroptosis inhibitors. **a** Flow chart of our screening strategy. **b** Cell viability of HT-1080 cells following treatment with RSL3 (0.5 μM) in the absence or presence of 4EGI-1 (10, 5, 2.5, 1.25 μM) or 4E1RCat (10, 5, 2.5, 1.25 μM) for 24 h. Mean ± SD, *n* = 3. **c** Cell viability of HT-1080 cells following treatment with RSL3 (0.5 μM), erastin (5 μM), ML162 (0.5 μM), ML210 (5 μM), FIN56 (2.5 μM), or FINO2 (10 μM) in the absence or presence of 4EGI-1 (10 μM) or 4E1RCat (10 μM) for 24 h. Mean ± SD, *n* = 3. **d** The accumulation of iron in HT-1080 and Calu-1 cells treated with RSL3 (0.5 μM) or erastin (5 μM) in the absence or presence of 4EGI-1 (10 μM) or 4E1RCat (10 μM) for 24 h. Mean ± SD, *n* = 3. **e** The accumulation of lipid hydroperoxides in HT-1080 and Calu-1 cells treated with RSL3 (0.5 μM, 4 h) or erastin (5 μM, 6 h) in the absence or presence of 4EGI-1 (10 μM) or 4E1RCat (10 μM). Mean ± SD, *n* = 3. **f**, **g** The antioxidant activity of the indicated compounds (10 μM) was analyzed by a 2,2-diphenyl-1-picrylhydrazyl (DPPH) or FENIX (STY-BODIPY) assay. Mean ± SD, *n* = 4; one-way ANOVA with Tukey’s multiple comparisons test. **h** The iron chelator activity of the indicated compounds (40 μM) was analyzed by using the ferrozine Fe^2+^ binding assay. Mean ± SD, *n* = 3; one-way ANOVA with Tukey’s multiple comparisons test. **i**, **j** Cell viability and intracellular glutathione (GSH) level of Calu-1 cells following treatment with RSL3 (0.5 μM) or erastin (5 μM) in the absence or presence of 4EGI-1 (10 μM), 4E1RCat (10 μM), or ferrostatin-1 (Fer-1, 1 μM) for 72 h. Mean ± SD, *n* = 3; two-way ANOVA with Tukey’s multiple comparisons test. **k** Immunoprecipitation (IP) analysis of EIF4E-binding proteins in Calu-1 cells following treatment with 4EGI-1 (10 μM) or 4E1RCat (10 μM) for 24 h. IB immunoblot. Mean ± SD, *n* = 3; one-way ANOVA with Tukey’s multiple comparisons test.

HT-1080 is a human sarcoma cell line that has been extensively used to study the mechanism and signaling pathways of ferroptosis^1^. The pharmacological inhibition of system xc^−^ or GPX4 by small molecule compounds (RSL3 and erastin) is a classic model for inducing ferroptotic cancer cell death^24^. Targeting the pathways of ferroptosis in tumor cells is an emerging anticancer strategy because malignant cells often rely on oncogenic or survival signals (e.g., iron accumulation, fatty acid synthesis, enhanced autophagic flux, and epithelial-mesenchymal transition) that render them particularly vulnerable to ferroptosis^25^. To further identify essential mediators of ferroptosis, we treated HT-1080 cells with the GPX4 inhibitor RSL3 in the absence or presence of a panel of 431 target-selective inhibitors (all used at 10 μM) for oncogenic signaling pathways (Supplementary Data 1). In addition to the previously reported lapatinib (epidermal growth factor receptor [EGFR]/ human epidermal growth factor receptor 2 [HER2] inhibitor), AZD8055 (the mechanistic target of rapamycin [MTOR] inhibitor), CH5132799 (MTOR/ phosphatidylinositol 3-kinase [PI3K] inhibitor), WZ4002 (EGFR inhibitor), and AICAR (AMP-activated protein kinase [AMPK] activator) with inhibitory activity on ferroptosis^26,27^ (Supplementary Data 1), this small-scale screening determined that 4EGI-1 and 4E1RCat were the two most effective inhibitors of the RSL3-induced growth inhibition of HT-1080 cells (Fig. 1a and Supplementary Data 1). Dose-response experiments confirmed the potential of 4EGI-1 and 4E1RCat to block the RSL3-induced death of human lung cancer Calu-1 cells, another common model used to study ferroptosis^1^ (Fig. 1b). The broad-spectrum protective activity of 4EGI-1 and 4E1RCat were corroborated in HT-1080 cells exposed to other ferroptosis inducers (including erastin, ML162, ML210, FIN56, and FINO2) (Fig. 1c).

We examined iron accumulation and lipid peroxidation, which are the two core events of ferroptosis^2^. 4EGI-1 and 4E1RCat failed to affect iron accumulation (Fig. 1d), but diminished lipid peroxidation (Fig. 1e) in HT-1080 and Calu-1 cells in response to RSL3 and erastin, suggesting that 4EGI-1 and 4E1RCat inhibit ferroptosis by interfering with downstream lipid peroxidation, instead of upstream iron accumulation. Unlike the effect of corresponding positive controls, including radical-trapping antioxidants (ferrostatin-1 and liproxstatin-1) and an iron chelator (deferoxamine), subsequent pharmaceutical chemical analysis (including the 2,2-diphenyl-1-picrylhydrazyl [DPPH] radical scavenging assay, fluorescence-enabled inhibited autoxidation [FENIX] assay, and the ferrozine Fe^2+^ binding assay) revealed that the anti-ferroptosis activity of 4EGI-1 and 4E1RCat was not related to any putative antioxidative (Fig. 1f, g) or iron-chelating abilities (Fig. 1h). Like ferrostatin-1, 4EGI-1 and 4E1RCat at 10 μM prevented erastin- or RSL3-induced ferroptosis in Calu-1 cells for 72 h (Fig. 1i). Compared to 4E1RCat, 4EGI-1 at 10 μM did not affect cell proliferation (Fig. 1i). 4E1RCat and 4EGI-1 had no effect on erastin-induced intracellular glutathione (GSH) depletion (Fig. 1j). In addition to HT-1080 and Calu-1 cells, 4E1RCat and 4EGI-1 inhibited erastin- or RSL3-induced cell death in PANC1 (a human pancreatic cancer cell line), HepG2 (a human liver cancer cell line), and mouse embryonic fibroblasts (MEF) (Supplementary Fig. 1a).

### EIF4E mediates ferroptosis independently from protein synthesis

**Fig. 2:**
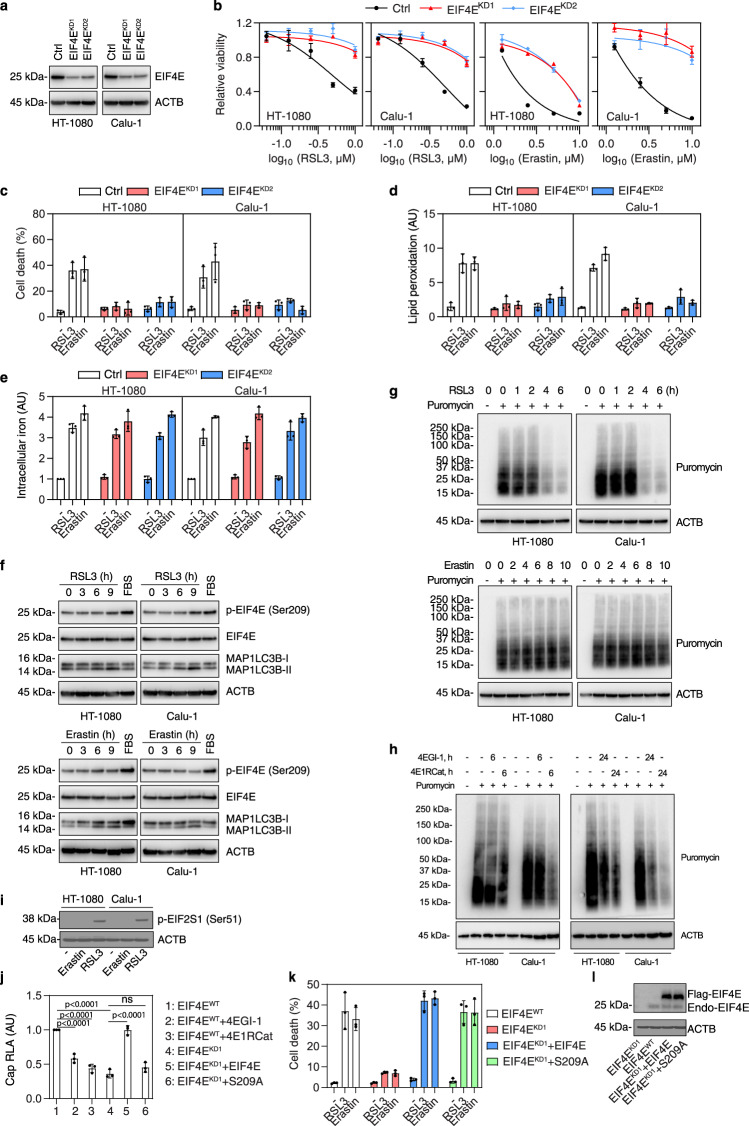
EIF4E mediates ferroptosis independent of protein synthesis. **a** Western blot analysis of the indicated proteins in control and EIF4E-knockdown (EIF4E^KD^) HT-1080 and Calu-1 cells. **b** Analysis of cell viability in the indicated cells following treatment with RSL3 or erastin for 24 h. Mean ± SD, *n* = 3. **c** Analysis of cell death in the indicated cells following treatment with RSL3 (0.5 μM) or erastin (5 μM) for 24 h. Mean ± SD, *n* = 3. **d** Analysis of lipid peroxidation in the indicated cells following treatment with RSL3 (0.5 μM, 4 h) or erastin (5 μM, 6 h). Mean ± SD, *n* = 3. **e** Analysis of iron accumulation in the indicated cells following treatment with RSL3 (0.5 μM) or erastin (5 μM) for 24 h. Mean ± SD, *n* = 3. **f** Western blot analysis of the indicated proteins in HT-1080 and Calu-1 cells following treatment with RSL3 (0.5 μM) or erastin (5 μM). FBS: medium having 20% fetal bovine serum for 9 h. **g**, **h** Western blot analysis of puromycylated peptides in HT-1080 and Calu-1 cells following treatment with RSL3 (0.5 μM), erastin (5 μM), 4EGI-1 (10 μM), or 4E1RCat (10 μM). The cells were incubated with or without puromycin (10 μg/ml) for 10 min prior to cell lysis. **i** Western blot analysis of the indicated proteins in HT-1080 and Calu-1 cells following treatment with RSL3 (0.5 μM) or erastin (5 μM) for 9 h. **j** The EIF4E-knockdown HT-1080 cells (EIF4E^KD1^) were transfected with wild-type (WT) EIF4E or EIF4E-S209A mutant along with cap-dependent luciferase reporter cDNA. 48 h after transfection, the transfected cells were treated with 4EGI-1 (10 μM) or 4E1RCat (10 μM) and harvested for luciferase assay (RLA). Mean ± SD, *n* = 3; one-way ANOVA with Tukey’s multiple comparisons test; ns not significant. **k** Analysis of cell death in indicated HT-1080 cells following treatment with RSL3 (0.5 μM) or erastin (5 μM) for 24 h. Mean ± SD, *n* = 3. **l** Western blot analysis of protein expression in indicated HT-1080 cells.

Both 4EGI-1 and 4E1RCat are pharmacological inhibitors of EIF4E, thereby reducing protein synthesis^28,29^. Consistent with previous studies^30,31^, immunoprecipitation analysis showed that 4EGI-1 (10 µM) increased the EIF4E-eukaryotic translation initiation factor 4E binding protein 1 (4EBP1) interaction in Calu-1 cells, while 4E1RCat (10 µM) inhibited the EIF4E-4EBP1 interaction (Fig. 1k). Both 4EGI-1 and 4E1RCat inhibited the binding of EIF4E to EIF4G1 (Fig. 1k), which is the structural partner of EIF4E in the translation initiation complex^19^. We next determined whether the genetic suppression of EIF4E expression also reduces the susceptibility to ferroptosis. The knockdown of EIF4E by two different shRNAs (Fig. 2a) limited RSL3- or erastin-induced growth inhibition (Fig. 2b), cell death (Fig. 2c), and lipid peroxidation (Fig. 2d), but did not inhibit iron accumulation (Fig. 2e), in HT-1080 and Calu-1 cells, corroborating a critical role for EIF4E in promoting ferroptosis. Moreover, transfection-enforced overexpression of EIF4E increased the sensitivity of cells to RSL3 or erastin (Supplementary Fig. 1b, c). In contrast, the overexpression of EIF4E inhibited the anticancer activity of the classic apoptosis inducer staurosporine (Supplementary Fig. 1d), which is consistent with the previously reported anti-apoptotic function of EIF4E^32^.

The protein synthesis pathways may promote or inhibit ferroptosis, depending on the context^33,34^. We determined whether EIF4E-mediated protein synthesis is essential for ferroptosis using four approaches. First, we employed the RiboLace, a method capable of examining the active translation of ribosome-associated RNAs^35^, to analyze the translatome of RSL3-induced cells under wildtype (WT) or EIF4E-knockdown conditions (Supplementary Data 2). Translatome profiling analysis did not find significant differences between WT and EIF4E-knockdown cells after RSL3 treatment (Supplementary Fig. 2a). Among 179 mRNAs that were significantly altered by RSL3 in WT cells, only 16 of them were observed in RSL3-treated EIF4E-knockdown cells (Supplementary Fig. 2b). Second, the knockdown of EIF4G1 by two different shRNAs failed to inhibit RSL3- or erastin-induced cell death in HT-1080 and Calu-1 cells (Supplementary Fig. 2c–e). As a control, the formation of the EIF4E-4EBP1 complex was increased in EIF4G1-knockdown HT-1080 cells (Supplementary Fig. 2f). Third, our investigation of the MAPK interacting serine/threonine kinase 1 (MKNK1, best known as MNK1)-mediated phosphorylation of EIF4E on Ser209, which enhances the function of EIF4E in protein synthesis^36^, suggests that EIF4E stimulates ferroptosis independently of its phosphorylation status. Unlike a positive control consisting of the addition of 20% fetal bovine serum (FBS), RSL3 and erastin failed to induce Ser209 phosphorylation of EIF4E (Fig. 2f). Consistently in previous studies^37,38^, RSL3 and erastin, but not 20% FBS, induced the accumulation of microtubule-associated protein 1 light chain 3 B-II (MAP1LC3B-II; a marker of autophagosomes) (Fig. 2f). The knockdown of MKNK1 had no effect on the anticancer activity of RSL3 and erastin in HT-1080 and Calu-1 cells (Supplementary Fig. 3a, b). As expected, in the absence or presence of 20% FBS, the knockdown of MKNK1 inhibited the phosphorylation of EIF4E on Ser209 (Supplementary Fig. 3c). Unlike 4EGI-1, the MKNK1 inhibitor CGP 57380 and the EIF4A inhibitor eFT226 failed to inhibit RSL3- or erastin-induced cell death in HT-1080 and Calu-1 cells (Supplementary Fig. 3d). Fourth, a puromycin labeling assay revealed that protein synthesis was inhibited in HT-1080 and Calu-1 cells by RSL3 (but not by erastin) (Fig. 2g). RSL3 was able to cause approximately 30% of cells to die within 6 h, most of them alive. As a positive control, 4EGI-1 and 4E1RCat inhibited protein synthesis in HT-1080 and Calu-1 cells (Fig. 2h). The phosphorylation of eukaryotic translation initiation factor 2 subunit alpha (EIF2S1, also known as eIF2α) on Ser51 can serve as an EIF4E-independent mechanism for inhibiting protein synthesis under certain conditions^39–42^. RSL3, rather than erastin, increased the phosphorylation of EIF2S1 in HT-1080 and Calu-1 cells (Fig. 2i). However, the EIF2S1 (S51A) mutant that cannot be phosphorylated at Ser51 (Supplementary Fig. 3e) had no significant effect on RSL3-induced cell death (Supplementary Fig. 3f). Fifth, EIF4E^S209A^ inhibited protein synthesis by measuring cap-dependent translation of luciferase reporter gene (Fig. 2j)^43^, but maintained the sensitivity of HT-1080 cells to erastin- or RSL3-induced cell death (Fig. 2k). As an internal control^28,44^, EIF4E inhibitors (4EGI-1 and 4E1RCat) did not block the hepatitis C virus (HCV) internal ribosome entry site (IRES)-mediated cap-independent translation initiation (Supplementary Fig. 3g). Western blotting confirmed that Flag-tagged wild-type and S209A EIF4E mutants had similar expression levels in indicated EIF4E-knockdown cells (Fig. 2l). Overall, these results indicate that EIF4E may favor ferroptosis in a protein synthesis-independent manner.

### The EIF4E-ALDH1B1 complex facilitates ferroptosis

**Fig. 3:**
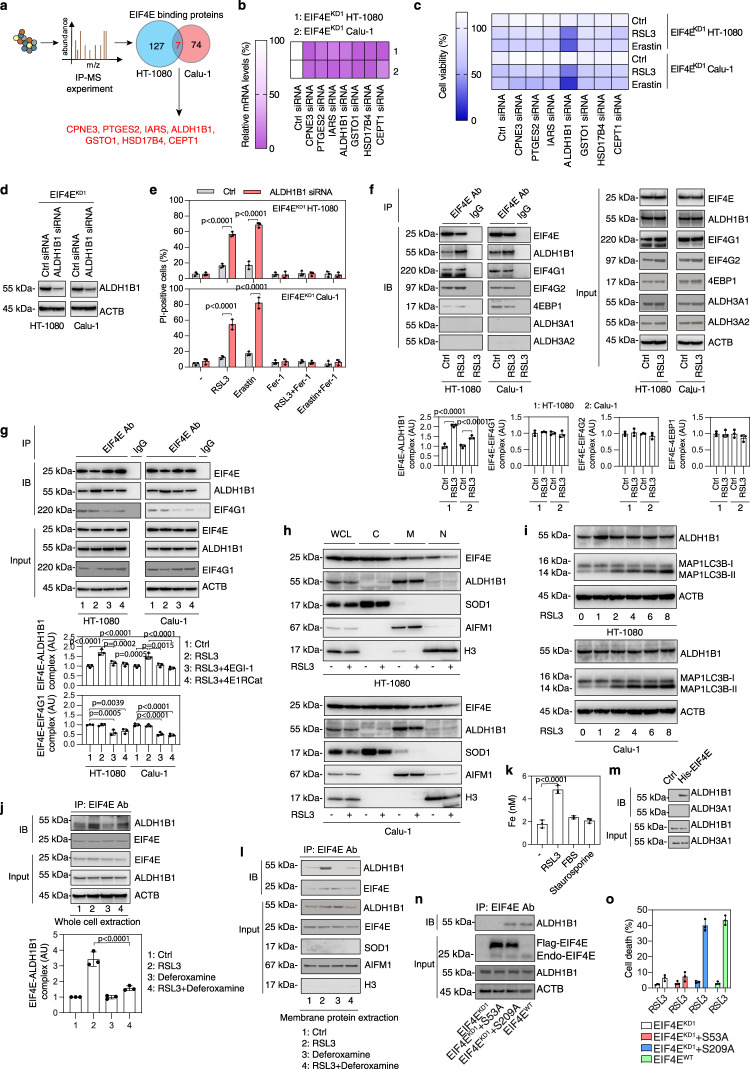
The EIF4E-ALDH1B1 complex facilities ferroptosis. **a** HT-1080 and Calu-1 cells were treated with RSL3 (0.5 μM, 4 h). EIF4E was immunoprecipitated using an anti-EIF4E antibody and subjected to mass spectrometry analysis. **b** Analysis of indicated mRNA in the corresponding siRNA knockdown EIF4E^KD^ HT-1080 and EIF4E^KD^ Calu-1 cells (*n* = 3). **c** Cell viability of indicated cells treated with RSL3 (0.5 μM) or erastin (5 μM) for 24 h (*n* = 3). **d** Western blot analysis of protein expression in indicated cells. **e** Cell death analysis of indicated cells treated with RSL3 (0.5 μM), erastin (5 μM), or ferrostatin-1 (Fer-1; 1 μM) for 24 h. Mean ± SD, *n* = 3; two-way ANOVA with Tukey’s multiple comparisons test. **f**, **g** Immunoprecipitation (IP) analysis of EIF4E-binding proteins in indicated cells treated with RSL3 (0.5 μM), 4EGI-1 (10 μM), or 4E1RCat (10 μM) for 4 h. Mean ± SD, *n* = 3; one-way ANOVA with Tukey’s multiple comparisons test. IB immunoblot. **h** Western blot analysis of whole-cell lysates (WCL) or cell fractionation (C: cytoplasmic; M: membrane/organelle; N: nuclear/cytoskeletal) of indicated cells treated with RSL3 (0.5 μM, 4 h). **i** Western blot analysis of the indicated proteins in indicated cells treated with RSL3 (0.5 μM) or erastin (5 μM) for the indicated time. **j**, **l** Immunoprecipitation (IP) analysis of EIF4E-ALDH1B1 complex in whole-cell extracts (**j**) or membrane protein extracts (**l**) of Calu-1 cells treated with RSL3 (0.5 μM) and/or deferoxamine (100 μM) for 4 h. Mean ± SD, *n* = 3; one-way ANOVA with Tukey’s multiple comparisons test. IB immunoblot. **k** Analysis of intracellular iron in Calu-1 cells treated with RSL3 (0.5 μM), 20% FBS, or staurosporine (0.25 μM) for 4 h. Mean ± SD, *n* = 3; one-way ANOVA with Tukey’s multiple comparisons test. **m** His-tag affinity pull-down analysis of the binding of recombinant protein EIF4E to ALDH1B1 or ALDH3A1. **n** Immunoprecipitation (IP) analysis of EIF4E-ALDH1B1 complex in indicated Calu-1 cells treated with RSL3 (0.5 μM, 4 h). IB immunoblot. **o** Cell death analysis of indicated Calu-1 cells treated with RSL3 (0.5 μM, 24 h). Mean ± SD, *n* = 3.

In addition to EIF4G1, EIF4E also binds to other proteins under certain conditions^45,46^. Using immunoprecipitation in combination with mass spectrometry, we examined the interactome of EIF4E during RSL3-induced ferroptosis (Supplementary Data 3). We compared our data with 130 known EIF4E-binding proteins reported in the protein–protein interaction database STRING (search tool for the retrieval of interacting genes/proteins)^47^. Regardless of the sample, 46 known EIF4E-binding proteins in the STRING database were confirmed in HT-1080 and Calu-1 cells (Supplementary Data 3). We further identified seven upregulated EIF4E-binding proteins that were at least twofold (including copine 3 [CPNE3], prostaglandin E synthase 2 [PTGES2], isoleucyl-tRNA synthetase 1 [IARS], ALDH1B1, glutathione S-transferase omega 1 [GSTO1], hydroxysteroid 17-beta dehydrogenase 4 [HSD17B4], and choline/ethanolamine phosphotransferase 1 [CEPT1]) in both HT-1080 and Calu-1 cells following RSL3 treatment (Fig. 3a). A subsequent RNAi screening showed that the depletion of ALDH1B1 (but not of any of the other 6 EIF4E-binding proteins) restored the sensitivity of EIF4E-knockdown cells (HT-1080 and Calu-1) to RSL3- or erastin-induced cell growth inhibition (Fig. 3b, c). A cell death assay further demonstrated that the ferroptosis sensitization of EIF4E-knockdown cells by ALDH1B1 depletion was blocked by ferrostatin-1 (Fig. 3d, e). In addition, genetic (by two different shRNAs) or pharmacological (using *N*,*N*-diethylaminobenzaldehyde [DEAB]) inhibition of ALDH1B1 enhanced, while the overexpression of ALDH1B1 attenuated, susceptibility to erastin or RSL3-induced ferroptosis in EIF4E wild-type cells (HT-1080 and Calu-1) (Supplementary Fig. 4a–g). 4EGI-1 and 4E1RCat failed to further protect ALDH1B1-knockout (ALDH1B1^−/−^) Calu-1 cells from RSL3- or erastin-induced ferroptosis (Supplementary Fig. 5a,b). The overexpression of ALDH1B1 reversed the increased susceptibility to RSL3- or erastin-induced ferroptosis in EIF4E-overexpressed Calu-1 cells (Supplementary Fig. 5c, d). Similarly, the overexpression of ALDH1B1 or the knockdown of EIF4E prevented ferroptosis caused by the inducible *Gpx4* deletion in Pfa1 cells (Supplementary Fig. 5e)^48^. These data suggest that EIF4E promotes ferroptosis by interacting with ALDH1B1.

We confirmed that an EIF4E-ALDH1B1 complex, but not the binding of EIF4E to EIF4G1, EIF4G2, or 4EBP1, was upregulated by RSL3 (Fig. 3f). Unlike the increase in the EIF4E-EIF4G1-EIF4G2 complex formation, there was no increase in the EIF4E-ALDH1B1 complex formation in HT-1080 cells following 20% FBS treatment (Supplementary Fig. 5f). Thus, EIF4E may form different stress protein complexes that regulate ferroptosis and translation initiation, respectively. The formation of the EIF4E-ALDH1B1 complex was not increased in response to the apoptosis inducer staurosporine (Supplementary Fig. 5f). Consistent with previous studies^49^, staurosporine inhibited the formation of the EIF4E-EIF4G1-EIF4G2 complex and increased the formation of the EIF4E-4EBP1 complex by inhibiting the phosphorylation of EIF4E on Ser209 (Supplementary Fig. 5f). EIF4E failed to bind to aldehyde dehydrogenase 3 family member A1 (ALDH3A1) or aldehyde dehydrogenase 3 family member A2 (ALDH3A2) (Fig. 3f), which is related to ferroptosis resistance under certain conditions^50,51^. However, EIF4E inhibitors (4EGI-1 and 4E1RCat) disrupted the RSL3-induced interaction between EIF4E and ALDH1B1 (Fig. 3g). Cell fractionation assays further showed that ALDH1B1 is mainly expressed in the membrane components (including organelle’s own membranes), while the subcellular distribution of EIF4E is widespread, including in the cell membrane, nucleus, and cytoplasm (Fig. 3h). The subcellular distribution of ALDH1B1 or EIF4E (Fig. 3h), as well as the expression levels of ALDH1B1 or EIF4E (Figs. 3i, 2f), were not significantly affected by RSL3 treatment.

Next, we investigated the effect of ferroptosis inducer signaling on the formation of the EIF4E-ALDH1B1 complex. The iron chelator deferoxamine inhibited RSL3-induced formation of the EIF4E-ALDH1B1 complex in Calu-1 cells (Fig. 3j). Compared with RSL3 treatment, 20% FBS and staurosporine did not increase intracellular iron accumulation (Fig. 3k). Furthermore, immunoprecipitation analysis revealed that deferoxamine inhibited RSL3-induced the formation of the ALDH1B1-EIF4E complex in membrane protein extraction (Fig. 3l), suggesting that iron accumulation, the most important initiation signal of ferroptosis^52^, is conducive to this process. The affinity-isolation assay demonstrated a direct interaction between EIF4E and ALDH1B1 recombinant protein (but not ALDH3A1 protein) (Fig. 3m). In contrast to the S209A mutant, which inhibits cap-dependent translation initiation^22^, the S53A mutant limits some of the nontranslational functions of EIF4E^53,54^. We revealed that the S209A mutant, rather than the S53A mutant, restored RSL3-induced formation of the EIF4E-ALDH1B1 complex and cell death in HT-1080 cells (Fig. 3n, o). We further observed that the EIF4E-ALDH1B1 complex was enriched in mitochondria (Supplementary Fig. 6a,b). Compared with the S209A mutant, the S53A mutant failed to restore RSL3-induced accumulation of EIF4E in mitochondria (Supplementary Fig. 6c). These findings indicate that the phosphorylation of EIF4E at Ser53 is necessary for the formation of the EIF4E-ALDH1B1 complex in mitochondria for ferroptosis.

### 4HNE is a product and mediator of ferroptosis

**Fig. 4:**
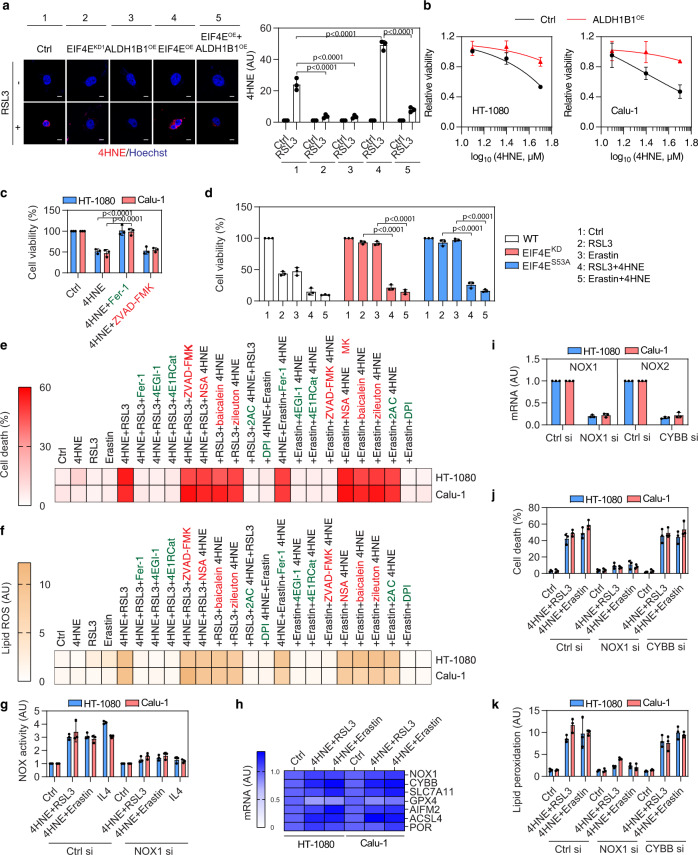
4HNE is a product and mediator of ferroptosis. **a** Immunofluorescence analysis showing staining of 4HNE in indicated Calu-1 cells treated with RSL3 (0.5 μM, 4 h) (scale bar = 10 µm). Mean ± SD, *n* = 3; two-way ANOVA with Tukey’s multiple comparisons test. **b** Cell viability of control and ALDH1B1-overexpressing (ALDH1B1^OE^) HT-1080 and Calu-1 cells treated with 4HNE for 24 h. Mean ± SD, *n* = 3. **c** Cell viability of ALDH1B1-knockdown HT-1080 and Calu-1 cells treated with 4HNE (50 μM) and/or ferrostatin-1 (Fer-1; 1 μM)/ZVAD-FMK (10 μM) for 24 h. Mean ± SD, *n* = 3; one-way ANOVA with Tukey’s multiple comparisons test. **d** Cell viability of indicated HT-1080 cells treated with RSL3 (0.5 μM) and erastin (5 μM) in the absence or presence of 4HNE (12.5 μM) for 24 h. Mean ± SD, *n* = 3; two-way ANOVA with Tukey’s multiple comparisons test. **e**, **f** Cell death (**e**, 24 h) or lipid peroxidation (**f**, 6 h) of indicated cells treated with 4HNE (12.5 μM), RSL3 (0.1 μM), and erastin (1 μM) in the absence or presence of ferrostatin-1 (Fer-1; 1 μM), 4EGI-1 (10 μM), 4E1RCat (10 μM), ZVAD-FMK (10 μM), necrosulfonamide (NSA; 1 µM), 2-acetylphenothiazine (2AC; 10 μM), diphenyleneiodonium (DPI; 1 μM), baicalein (10 μM), or zileuton (10 μM) (*n* = 3). **g** NOX activity of indicated cells treated with IL4 (50 ng/ml) or 4HNE (12.5 μM) plus RSL3 (0.1 μM) or erastin (1 μM) for 6 h. Mean ± SD, *n* = 3. **h** Analysis of gene expression in indicated cells treated with 4HNE (12.5 μM) in the presence of RSL3 (0.1 μM) or erastin (1 μM) for 24 h (*n* = 3). **i** Analysis of NOX1 and CYBB/NOX2 gene expression in indicated cells. Mean ± SD, *n* = 3. **j** Cell death of indicated cells treated with 4HNE (12.5 μM) plus RSL3 (0.1 μM) or erastin (1 μM) for 24 h. Mean ± SD, *n* = 3. **k** Lipid peroxidation of indicated cells treated with 4HNE (12.5 μM) plus RSL3 (0.1 μM, 4 h) or erastin (1 μM, 6 h). Mean ± SD, *n* = 3.

Studies have shown that 4HNE is the main aldehyde produced during the lipid peroxidation of PUFAs and serves as one of the biomarkers of ferroptosis^55^. However, it is unclear whether increased 4HNE production might affect feedback circuitries to reduce the sensitivity of cells to ferroptosis. The ALDH family possesses different activities in catalyzing the oxidation of highly toxic 4HNE to the less toxic 4-hydroxynonenoic acid^56^. ALDH1B1 has previously been reported to metabolize 4HNE, albeit with low affinity for its binding and poor catalytic efficiency in cell-free systems^57,58^. We found that the overexpression of ALDH1B1 limited the accumulation of 4HNE in WT and EIF4E-overexpressed Calu-1 cells following treatment with RSL3 (Fig. 4a), suggesting that ALDH1B1 still plays an important role in limiting 4HNE production in cells during ferroptosis. Previous studies showed that 4HNE at 25–100 μM can cause significant cell death^59,60^. Consistently, overexpressed ALDH1B1 limited the growth inhibition induced by the addition of high doses of exogenous 4HNE in HT-1080 and Calu-1 cells (Fig. 4b). A ferroptosis inhibitor (ferrostatin-1), but not a caspase inhibitor (ZVAD-FMK), inhibited the cytotoxicity caused by 4HNE (50 μM) in ALDH1B1-knockdown HT-1080 and Calu-1 cells (Fig. 4c). 4HNE treatment also restored the sensitivity of EIF4E-knockdown cells and EIF4E-S53A cells to RSL3- or erastin-induced cell growth inhibition (Fig. 4d). These results confirm the importance of the EIF4E-ALDH1B1 complex in regulating 4HNE accumulation and toxicity during ferroptosis.

Next, we sought to investigate whether a subtoxic concentration of 4HNE (12.5 µM) affects susceptibility to ferroptosis. In fact, low-dose 4HNE enhanced low-dose RSL3- or erastin-induced cell death (Fig. 4e) and lipid peroxidation (Fig. 4f), and these processes were reversed by the administration of ferrostatin-1, 4EGI-1, or 4E1RCat (but not ZVAD-FMK and necrosulfonamide [an inhibitor of necroptosis]) in HT-1080 and Calu-1 cells. These analyses indicate that even low levels of 4HNE can accelerate the development of RSL3- or erastin-induced lipid peroxidation and subsequent ferroptosis.

The putative function of 4HNE in amplifying ferroptosis is to induce the activation of NOXs (e.g., NADPH oxidase 1 [NOX1] and cytochrome B-245 beta chain [CYBB/NOX2])^61^, which are membrane enzymes that produce ROS in ferroptosis to initiate lipid peroxidation. The NOX activity was increased by low-dose 4HNE in the presence of RSL3 or erastin (Fig. 4g). As a positive control^62^, the knockdown of NOX1 inhibited interleukin 4 (IL4)-induced the upregulation of NOX1 activity (Fig. 4g). 4HNE + RSL3 or 4HNE + erastin had no significant effects on the mRNA expression of NOX1, CYBB, and other ferroptosis regulators (such as solute carrier family 7 member 11 [SLC7A11], GPX4, AIFM2, acyl-CoA synthetase long-chain family member 4 [ACSL4], and POR) (Fig. 4h). Additionally, 2-acetylphenothiazine (2AC, a potent and selective NOX1 inhibitor) or diphenyleneiodonium (DPI, a pan-NOX inhibitor) blocked 4HNE-induced cell death and lipid peroxidation in the presence of RSL3 or erastin, but ALOX inhibitors (baicalein and zileuton) failed to affect the system (Fig. 4e, f). The DPPH and FENIX assays revealed that 2AC was not a radical-trapping antioxidant (Fig. 1f, g). The knockdown of NOX1, but not of CYBB (Fig. 4i), by siRNAs inhibited 4HNE-induced cell death and lipid peroxidation in the presence of RSL3 or erastin (Fig. 4j, k). Similarly, the knockout of NOX1 (NOX1^−/−^) by CRISPR-Cas9 gene editing prevented ferroptosis caused by 4HNE with RSL3 or erastin, and this process was not enhanced by 4EGI-1 or 4E1RCat (Supplementary Fig. 7a,b). DEAB-mediated sensitivity to RSL3 or erastin was also inhibited in NOX1^−/−^ cells (Supplementary Fig. 7c). Moreover, the knockdown of NOX1 reversed the increased susceptibility to ferroptosis in EIF4E-overexpressed or ALDH1B1-knockdown Calu-1 cells (Supplementary Fig. 7d, e). Together, these data support the hypothesis that the EIF4E-ALDH1B1-4HNE pathway induces ferroptosis by activating NOX1, but not ALOX or CYBB.

### The EIF4E-ALDH1B1-4HNE axis regulates ferroptosis sensitivity in vivo

**Fig. 5:**
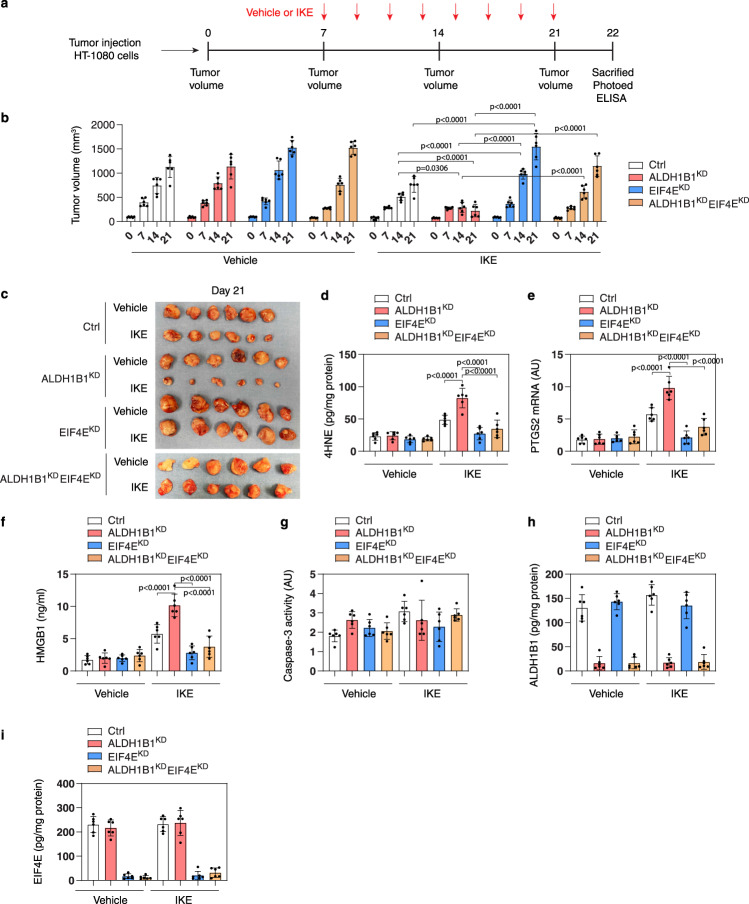
EIF4E and ALDH1B1 regulates ferroptosis sensitivity in vivo. **a** A scheme of treatment in an HT-1080 xenograft tumor model with IKE. Athymic nude mice were injected subcutaneously with indicated ALDH1B1-knockdown (ALDH1B1^KD^) or EIF4E-knockdown (EIF4E^KD^) HT-1080 cells for 7 days and then treated with IKE (40 mg/kg, i.p., once every other day) at day 7 for 2 weeks. **b** Tumor volumes were calculated weekly (*n* = 6 mice/group; two-way ANOVA with Tukey’s multiple comparisons test; data are presented as mean ± SD). **c** Photographs of isolated tumors on day 14 after treatment. **d**–**i** The levels of 4HNE (**d**) and PTGS2 mRNA (**e**) in isolated tumors, serum HMGB1 (**f**), caspase-3 activity (**g**), ALDH1B1 protein (**h**), and EIF4E protein (**i**) in isolated tumors at day 14 after treatment were assayed (*n* = 6 mice/group; two-way ANOVA with Tukey’s multiple comparisons test; data were presented as mean ± SD).

Imidazole ketone erastin (IKE) is an erastin analog with improved potency and metabolic stability that is suitable for animal studies^63^. We determined whether the EIF4E-ALDH1B1 axis regulates the anticancer activity of IKE in xenograft mouse models. Human EIF4E-knockdown, ALDH1B1-knockdown, or control HT-1080 cells were implanted subcutaneously into the left flank of immunodeficient nude mice. One week later, tumor-bearing mice were intraperitoneally injected with IKE (40 mg/kg/day for 2 weeks) (Fig. 5a). Compared with its effect on the control group, IKE-mediated tumor suppression was weakened in the EIF4E-knockdown group, but enhanced in the ALDH1B1-knockdown group (Fig. 5b, c). During IKE treatment, double knockdown of EIF4E/ALDH1B1 reversed the phenotype of the ALDH1B1-knockdown group (Fig. 5b, c). Quantitation of 4HNE and prostaglandin-endoperoxide synthase 2 (PTGS2; a biomarker of ferroptosis in vivo^12^) in tumors, as well as the concentration of high-mobility group box 1 (HMGB1; a DAMP molecule involved in ferroptotic cell death^64^) in serum, further supports the hypothesis that EIF4E promotes and ALDH1B1 inhibits IKE-induced ferroptosis in vivo (Fig. 5d–f). As a control, the caspase-3 activity in tumor tissue was not changed by IKE in the absence or presence of EIF4E or ALDH1B1 (Fig. 5g). As expected, ELISA analysis showed that the protein levels of ALDH1B1 or EIF4E were downregulated in the ALDH1B1^KD^ or EIF4E^KD^ groups, respectively (Fig. 5h, i).

Compared with the EIF4E^KD^ group, the anticancer activity of IKE in the EIF4E^KD^NOX1^OE^ group was restored (Supplementary Fig. 8a–f). In contrast, compared with the EIF4E^OE^ group, the anticancer activity of IKE in the EIF4E^OE^NOX1^KD^ group was limited (Supplementary Fig. 8g–l). These animal studies further demonstrated that NOX1 is a downstream effector of EIF4E-mediated ferroptosis caused by IKE.

**Fig. 6:**
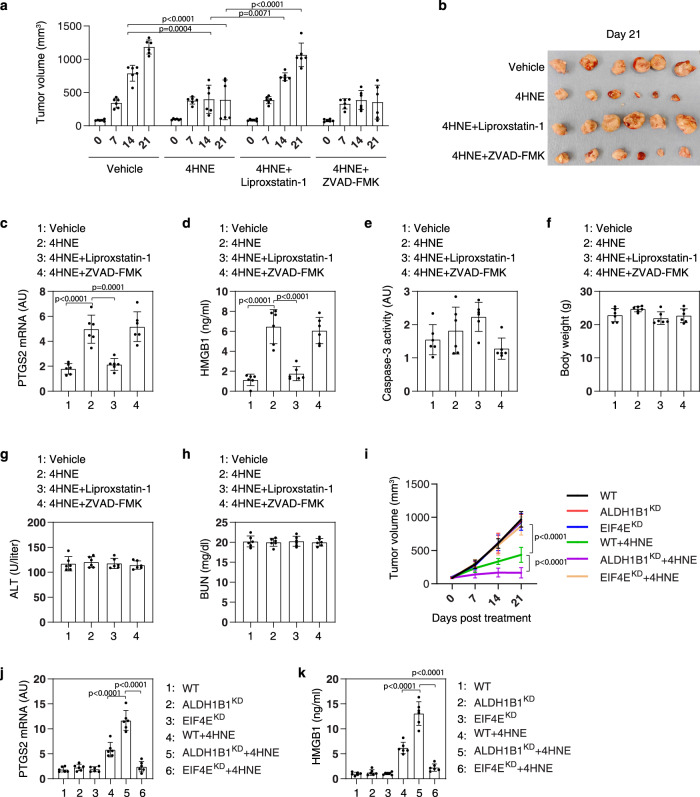
4HNE suppresses tumor growth by inducing ferroptosis in vivo. **a** Athymic nude mice were injected subcutaneously with HT-1080 cells for 7 days and then given intratumoral treatment with 4HNE (5 mg/kg, once every other day) in the absence or presence of ZVAD-FMK (5 mg/kg, once every other day) or liproxstatin-1 (5 mg/kg, once every other day) at day 7 for 2 weeks. Tumor volumes were calculated weekly (*n* = 6 mice/group; two-way ANOVA with Tukey’s multiple comparisons test; data were presented as mean ± SD). **b** Photographs of isolated tumors on day 14 after treatment. **c**–**h** The levels of PTGS2 mRNA (**c**), serum HMGB1 (**d**), and caspase-3 activity in isolated tumors (**e**); body weight (**f**), serum ALT (**g**), and serum BUN (**h**) at day 14 after treatment were assayed (*n* = 6 mice/group; one-way ANOVA with Tukey’s multiple comparisons test; data were presented as mean ± SD). **i** Athymic nude mice were injected subcutaneously with indicated HT-1080 cells for 7 days and then given intratumoral treatment with 4HNE (5 mg/kg, once every other day) at day 7 for 2 weeks. Tumor volumes were calculated weekly (*n* = 6 mice/group; two-way ANOVA with Tukey’s multiple comparisons test; data are presented as mean ± SD). **j**, **k** The levels of PTGS2 mRNA in tumor (**j**) and serum HMGB1 (**k**) at day 14 after treatment were assayed (*n* = 6 mice/group; one-way ANOVA with Tukey’s multiple comparisons test; data were presented as mean ± SD).

We also evaluated the effects of high-dose 4HNE (5 mg/kg) on tumor suppression in vivo. The local intratumoral injection of 4HNE inhibited the growth of HT-1080 tumors, and this effect was reversed by simultaneous instillation of liproxstatin-1 (but not by ZVAD-FMK), suggesting that high-dose 4HNE can trigger ferroptosis in vivo (Fig. 6a, b). As expected, the levels of the ferroptosis biomarker PTGS2 in tumors and those of circulating HMGB1 were increased by 4HNE (Fig. 6c, d). In contrast, caspase-3 activity was not affected by high-dose 4HNE (Fig. 6e). To evaluate the toxicity of 4HNE in nude mice, their body weights, serum alanine aminotransferase (ALT), and serum blood urea nitrogen (BUN) were measured. There were no differences in body weight, ALT, and BUN between the 4HNE-treated group and the control group (Fig. 6f–h). These findings suggest that a local supply of 4HNE can suppress tumor growth by inducing ferroptosis without any systemic side effects. 4HNE-mediated tumor suppression was reduced in the EIF4E-knockdown group, but enhanced in the ALDH1B1-knockdown group (Fig. 6i). Quantitation of PTGS2 in tumors and HMGB1 in serum further supported the hypothesis that EIF4E promotes and ALDH1B1 blocks 4HNE-induced ferroptosis in vivo (Fig. 6j, k).

## Discussion

**Fig. 7:**
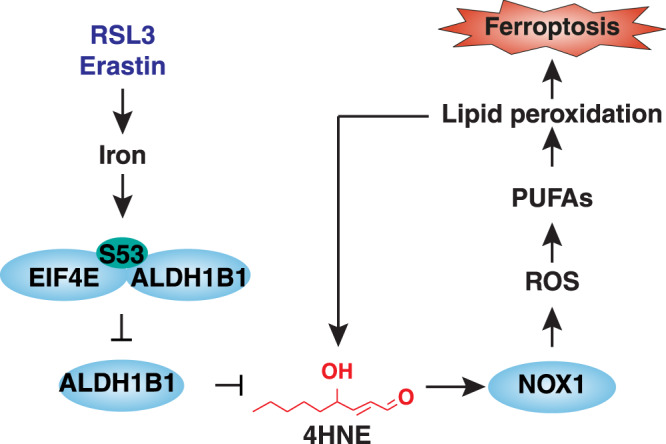
A model illustrating the function of EIF4E and ALDH1B1 in regulating 4HNE-mediated ferroptosis. EIF4E promotes ferroptosis by directly blocking ALDH1B1, thereby promoting the accumulation of toxic 4HNE and subsequent NOX1-dependent lipid peroxidation.

Compared to their normal counterparts, cancer cells show elevated intracellular ROS levels and correspondingly enhanced antioxidant capacity^65^. Therefore, it can be expected that robust activation of ROS production would cause a redox imbalance and selectively kill cancer cells^66^. However, chronic oxidative stress increases genomic instability and triggers inflammatory responses, favoring the formation and progression of tumors^67^. Accordingly, ferroptosis, an oxidative cell death modality, plays a dual role in oncogenesis. Understanding the process and function of ferroptotic death is certainly important for the development of new anticancer strategies. Here, we unveiled an unexpected role of EIF4E in ferroptosis by directly blocking ALDH1B1, thereby promoting the accumulation of toxic 4HNE (Fig. 7). This pro-ferroptosis function of EIF4E is different from its well-known pro-survival activity of protein synthesis^20,21^.

Mechanistically, EIF4E apparently performs its functions in ferroptosis and protein synthesis in the context of distinct protein complexes. Ferroptosis mediated by EIF4E does not require the traditional EIF4E-EIF4G1 translation initiation complex activated by its upstream kinase MKNK1 and subsequent phosphorylation of EIF4E at Ser209. In contrast, phosphorylation of EIF4E at Ser53 mediates the formation of the EIF4E-ALDH1B1 complex, which contributes to iron-dependent ferroptosis. Although upregulated EIF4E is well characterized in promoting tumor growth, we provide evidence that EIF4E has a tumor suppressor function in HT-1080 and Calu-1 cells as it binds to ALDH1B1 to induce ferroptosis. Whether the EIF4E-ALDH1B1 complex plays a similar role in inhibiting tumor growth of other cancer cells requires further investigation in the future. Regardless, understanding the mutation and binding partner status of EIF4E is important for precision oncology medicine.

In addition to the plasma membrane, other membrane-delimited organelles, such as the endoplasmic reticulum^34^, Golgi apparatus^68^, lysosomes^69–71^, mitochondria^72^, lipid droplets^73^, and peroxisomes^74^, play a context-dependent role in modulating ferroptosis sensitivity. It is possible that the EIF4E-ALDH1B1 complex is enriched mitochondria-associated membranes to mediate ferroptosis. In contrast, dihydroorotate dehydrogenase (DHODH) plays an important role in inhibiting mitochondrial membrane lipid peroxidation during ferroptosis^75^. Although ferroptosis was initially considered as being unrelated to autophagy^1^, accumulating data suggest that unrestricted autophagy promotes ferroptosis by promoting iron overload or lipid peroxidation^37,38,73,76–80^. Thus, it is possible that autophagic signals are generated in response to ferroptotic stress and activate iron accumulation and, subsequently, the EIF4E-ALDH1B1 complex, but this remains to be investigated.

Our findings highlight a role for ALDH1B1 in the inhibition of ferroptosis through lipid detoxification. While some ALDH family members are known to oxidize 4HNE and other aldehydes to their corresponding acids, only recently have ALDH3A1 and ALDH3A2 been recognized as being associated with ferroptosis resistance in head and neck cancers, gastric tumors, and leukemia cells^50,51^. In the current study, we provide genetic evidence that the expression of ALDH1B1 suppresses 4HNE-mediated ferroptosis. Strikingly, ALDH1B1 not only inhibits 4HNE accumulation, but also blocks 4HNE-triggered lipid peroxidation, implying that the ALDH family may serve as a hub to relieve the production and activity of toxic lipid metabolites during ferroptosis. Thus, ALDH enzymes may constitute an alternative to the endosomal sorting complex required for transport-III (ESCRT-III)-dependent membrane repair machinery in mitigating membrane damage during ferroptosis^81,82^.

While 4HNE is a major aldehyde byproduct during lipid peroxidation, details are lacking on the mechanisms by which 4HNE promotes ferroptotic signaling. Here, we propose a model in which 4HNE accumulation leads to the selective activation of NOX1 (instead of CYBB and ALOXs), thus causing lipid peroxidation and subsequent ferroptotic death. This is consistent with previous studies showing that NOX1 favors ferroptosis in certain cancer cells^10^. NOX1 is unlikely to be the only mediator of 4HNE-mediated cytotoxicity, because 4HNE can cause covalent modification of many proteins, lipids, and DNA^83^. Regardless, the 4HNE-NOX1 pathway may engage in a positive feedback loop with lipid peroxidation, thereby accelerating ferroptosis. We demonstrate that 4HNE is not only a product of lipid peroxidation, but also forms positive feedback to accelerate lipid peroxidation through the activation of NOX1. This 4HNE-dependent process can also be blocked by ferrostatin-1.

Despite our proof that the EIF4E-ALDH1B1-NOX1 pathway regulates ferroptosis in cancer cells, the physiological role of EIF4E-dependent ferroptosis remains undetermined. Meanwhile, mouse models only partially mimic the pathological and clinical features of human tumors. The significance of activating EIF4E-dependent ferroptosis for tumor treatment needs to be further verified in human clinical trials.

In summary, EIF4E mediates ferroptosis by directly inhibiting ALDH1B1, which is a key regulator for quenching 4HNE. This previously unrecognized metabolic framework of lipid toxicity may be useful for designing cancer treatments.

## Methods

The reagents are described in Supplementary Table 1.

### Cell culture

HT-1080 (CCL-121), Calu-1 (HTB-54), PANC1 (CRL-1469), HepG2 (HB-8065), and MEF (SCRC-1008) cell lines were obtained from the American Type Culture Collection. EIF2S1^S51A^ cells were created as previously described^84^. These cell lines were grown in Dulbecco’s modified Eagle’s medium (DMEM) or RPMI-1640 medium with 10% fetal bovine serum, 2 mM l-glutamine, and penicillin and streptomycin (100 U/ml). All cells were mycoplasma-free and authenticated using short tandem repeat DNA profiling analysis.

### Drug screening and cell viability assay

Cells were seeded at 1 × 10^4^ cells per well into 96-well plates and incubated with a panel of 431 target-selective inhibitors (each 10 µM) or indicated drugs in triple wells. Subsequently, 100 μl of fresh medium was added to cells containing 10 μl of Cell Counting Kit (CCK)-8 solutions (Bimake, B34304) and incubated for 1 h in 5% CO_2_ at 37 °C. Absorbance at 450 nm was measured using a microplate reader (Cytation 5 Cell Imaging Multi-Mode Reader). Dimethyl sulfoxide (DMSO) was used to prepare the stock solution of drugs. The final concentration of DMSO in the drug-working solution in the cells was <0.01%. DMSO of 0.01% was used as vehicle control in all cell culture assays.

### Lipid peroxidation assay

Cells were seeded at 1 × 10^5^ cells per well into 12-well plates and incubated with the indicated treatments in 5% CO_2_ at 37 °C. During the last 30 min of incubation, 1 μg/ml of Hoechst 33342 (Thermo Fisher Scientific, 62249) and 10 μM BODIPY 581/591 C11 (Thermo Fisher Scientific, D3861) dyes were added. After the cells were washed with PBS, they were imaged using an EVOS imaging system (Thermo Fisher Scientific). Image analysis was conducted with Image J software (version 1.52 v) based on 8–10 random fields. The relative lipid peroxidation was quantified by using the ratio of green fluorescence intensity (BODIPY 581/591 C11-oxidized) to red fluorescence intensity (BODIPY 581/591 C11-reduced)^26^.

### DPPH assay

We dissolved 2,2-diphenyl-1-picrylhydrazyl (DPPH; Sigma-Aldrich, D9132) in methanol to a final concentration of 100 μM. The tested compounds were added to 1 ml of DPPH solution with a final concentration of 10 μM. Samples were mixed well and incubated at room temperature for 1 h. The absorbance at 517 nm (indicating the concentration of nonreduced DPPH) was measured using a microplate reader (Cytation 5 Cell Imaging Multi-Mode Reader). Results were normalized to DMSO (which has no antioxidant activity; set as 100%).

### FENIX assay

The fluorescence-enabled inhibited autoxidation (FENIX) microplate-based assay was performed according to the previous protocol^85^. In brief, egg phosphatidylcholine liposomes (1 mM), STY-BODIPY (1 µM), and the indicated compounds (10 μM) were vortexed in PBS (pH7.4), then 297 µl aliquots of the mixture were incubated in black 96-well polypropylene plate in a BioTek Synergy H1 plate reader at 37 °C for 10 min. The plate was ejected from the plate reader and the autoxidation was initiated by adding 3 µL aliquots of (2,2-azobis(2-amidinopropane)dihydrochloride (AAPH, 1 mM), followed by a mixing protocol for 5 min. The kinetic data of [STY-BODIPYox] was acquired by excitation of the probes at 488 nm and emission was measured at 518 nm.

### Ferrozine iron chelation assay

Iron (II) chloride (Sigma-Aldrich, 372870) was dissolved in water to a final concentration of 10 μM. The tested compounds were added to 1 ml of iron (II) chloride solution with a final concentration of 40 μM and incubated at room temperature for 10 min. Next, 3-(2-pyridyl)-5,6-diphenyl-1,2,4-triazine-p,p′-disulfonic acid monosodium salt hydrate (FerroZine Iron Reagent; Sigma-Aldrich, 160601) was added and mixed with a final concentration of 20 μM. Samples were mixed well and incubated at room temperature for 1 h. The absorbance at 562 nm was measured using a microplate reader. Results were normalized to DMSO (which has no iron-chelating activity; set as 100%).

### NOX activity assay

NOX activity was measured using the lucigenin-enhanced chemiluminescence method^10^. Briefly, cultured cells were homogenized in lysis buffer (20 mM KH_2_PO_4_, pH 7.0, 1 mM EGTA, 1 mM phenylmethylsulfonyl fluoride, 10 µg/ml aprotinin, and 0.5 µg/ml leupeptin) by using a Dounce homogenizer (100 strokes on ice). Homogenates were centrifuged at 800 × *g* at 4 °C for 10 min to remove the unbroken cells and debris, and aliquots were used immediately. To start the assay, 100 µl aliquots of homogenates were added to 900 µl of 50 mM phosphate buffer, pH 7.0, containing 1 mM EGTA, 150 mM sucrose, 5 µM lucigenin, and 100 µM NADPH. Photon emission in terms of relative light units was measured in a luminometer every 30 s for 5 min. There was no measurable activity in the absence of NADPH. NOX activity was expressed as relative activity and normalized to protein concentration.

### Cell death assay

Cells were seeded at a density of 2 × 10^5^ cells/well in DMEM medium in six-well plates. The next day, cells were incubated with the indicated treatments. After that, the cells were then stained with propidium iodide (Thermo Fisher Scientific, R37108) for 30 min in an incubator of 5% CO_2_ at 37 °C. Morphological changes were examined by fluorescence microscope at ×20 magnification. A Countess II FL Automated Cell Counter (Thermo Fisher Scientific) was used to assay the percentages of dead cells after propidium iodide staining.

### Puromycylation assay

Cells were seeded at a density of 2 × 10^5^ cells/well in DMEM medium in 6-well plates. The next day, cells were incubated with the indicated treatments. After that, treatment media was replaced with DMEM medium containing 10 μg/ml puromycin (InvivoGen, ant-pr-1) and labeled for 15 min in an incubator of 5% CO_2_ at 37 °C. At this point, cells were harvested and processed via western blot. The primary antibody was mouse anti-puromycin at a dilution of 1:4000 (Sigma-Aldrich, MABE343, clone 12D10).

### RNA interference, gene transfection, and gene editing

The transfection of shRNA, siRNA, or cDNA was performed with Lipofectamine 3000 (Thermo Fisher Scientific, L3000-015) according to the manufacturer’s protocol. For the transfection of shRNA, 293FT cells (Thermo Fisher Scientific, R70007) were used to produce high-titer lentiviral particles, and the virus-containing medium was harvested 48 h after transfection. RNAi was performed using lentiviral transduction, as previously described in ref. 86. Puromycin (5 μg/ml; InvivoGen, ant-pr-1) was used for the selection of transduced cells. CRISPR-Cas9-mediated gene editing was performed in close adherence to Feng Zhang lab’s protocol^87^. The sequence or order information of shRNA, siRNA, gRNA, and cDNA are shown in Supplementary Table 1.

### qPCR analysis

Total RNA was extracted and purified from cultured cells using the RNeasy Plus Mini Kit (QIAGEN, 74136). First-strand cDNA was synthesized from 1 µg of RNA using the iScript cDNA Synthesis Kit (Bio-Rad, 1708890). Briefly, 20-µl reactions were prepared by combining 4 µl of iScript Select reaction mix, 2 µl of gene-specific enhancer solution, 1 µl of reverse transcriptase, 1 µl of gene-specific assay pool (20×, 2 µM), and 12 µl of RNA diluted in ribonuclease-free water. The cDNA from various cell samples was then amplified by real-time qPCR with specific primers using the CFX96 Touch Real-Time PCR Detection System (Bio-Rad) with the CFX Manager Software (Bio-Rad, version 3.1). The gene expression was calculated via the 2^−ΔΔCt^ method and normalized to *18SRNA*. The relative concentrations of mRNA were expressed in arbitrary units based on the untreated group, which was assigned a value of 1. The primers, which were synthesized and desalted from Sigma-Aldrich, are shown in Supplementary Table 1.

### Cell fractionation

Cell Fractionation Kit (Cell Signaling Technology, 9038) was used for cell fractionation according to the manufacturer’s protocol. Whole-cell lysates (WCL) were used to represent total protein. Cytoplasmic proteins (C) were isolated using CIB buffer. Integral membrane and organellular membrane proteins (M), including mitochondrial membrane proteins, were isolated using MIB buffer. Nuclear proteins and cytoskeletal proteins (N) were isolated using CyNIB buffer. All buffers were provided in the Cell Fractionation Kit. Western blot analysis of cell fractions from indicated cells using SOD1 (Abcam,ab16831, 1:1000), AIFM1/AIF (Cell Signaling Technology, 5318, 1:1000), Histone H3 antibody (Cell Signaling Technology, 9715, 1:1000) showing cytoplasmic, membrane, and nuclear localization.

Mitochondria-associated membranes (MAMs) were isolated by sequential centrifugation-based fractionation as previously published^88^. In brief, cells (10^9^) were homogenized with a Dounce homogenizer (100 strokes) in homogenization buffer (225 mM mannitol, 75 mM sucrose, 30 mM Tris-HCl pH 7.4, 0.1 mM ethylene glycol-bis(β-aminoethylether)-*N*,*N*,*N*′,*N*′-tetraacetic acid [EGTA], and PMSF [phenylmethylsulfonyl fluoride]) and then centrifuged at 740 × *g* for 5 min to remove entire cells. The supernatant was then centrifuged at 9000 × *g* for 10 min to pellet the crude mitochondrial fraction. Crude mitochondria were resuspended in 0.5 ml mitochondrial re-suspension buffer (MRB; 250 mM mannitol, 5 mM HEPES [pH 7.4] and 0.5 mM EGTA) and layered on top of 30% Percoll (8 ml) followed by MRB (4 ml). After centrifugation at 95,000 × *g* for 30 min, the MAM band was aspirated from the gradient. The MAM was diluted fivefold and centrifuged at 9000 × *g* for 10 min to remove mitochondrial contamination. To pellet the MAM, the supernatant was centrifuged at 100,000 × *g* for 1 h. All centrifugation steps were performed at 4 °C.

### Western blot analysis

Cells or subcellular components were lysed three times with 1× cell lysis buffer (Cell Signaling Technology, 9803) containing protease inhibitor on ice for 10 min. Protein was quantified using a bicinchoninic acid (BCA) assay (Thermo Fisher Scientific, 23225), and 20 to 40 µg of each sample was resolved on 4 to 12% Criterion XT Bis-Tris gels (Bio-Rad, 3450124) in XT MES running buffer (Bio-Rad, 1610789) and transferred to polyvinylidene difluoride membranes (Bio-Rad, 1620233) using the Trans-Blot Turbo Transfer Pack and System. Membranes were blocked with tris-buffered saline with Tween 20 (TBST) containing 5% skim milk for 1 h and incubated overnight at 4 °C with various primary antibodies. Following three washes in TBST, membranes were incubated with goat anti-rabbit or anti-mouse immunoglobulin G (IgG) horseradish peroxidase secondary antibody (Cell Signaling Technology, 7074 or 7076; 1:1000) at room temperature for 1 h and washed. Chemiluminescence substrate was applied using the SuperSignal West Pico Chemiluminescent Substrate (Thermo Fisher Scientific, 34080) or the SuperSignal West Femto Maximum Sensitivity Substrate (Thermo Fisher Scientific, 34095), and blots were analyzed using the ChemiDoc Touch Imaging System (Bio-Rad) and Image Lab Software (Bio-Rad, version 6.1)^77^. The information on antibodies is shown in Supplementary Table 1.

### RiboLace

RiboLace were performed using the Ribosome Profiling All-In-One Set Kit (Immagina BioTechnology, RS-001s) according to the manufacturer’s protocol. Briefly, cells were lysed with the lysis buffer (1% sodium deoxycholate, 5 U/mL DNase I, and 200 U/mL RiboLock RNase inhibitor). The digested cell lysate was added to the functionalized beads and incubated for 70 min at 4 °C with orbital rotation on a wheel at 3 rpm. The tubes were then kept on ice on a magnetic rack for 5 min to pellet the bead-bound ribosomes. The supernatant was separated, and beads were washed twice with 500 µl of W-buffer and then resuspended in 200 µl W-buffer. After adding 20 µL of 1% SDS and 5 µL of proteinase K and incubating in a 37 °C water bath for 75 min, RNA was extracted from the bead-bound ribosomes using the phenol/chloroform example protocol and resuspended in 5 µL of nuclease-free water. The ribosome-protected RNA fragments were converted into a DNA library for sequencing at the UT Southwestern Genomics Core. Cluster analysis was performed based on the similarity of variables or their opposite distances.

### Immunoprecipitation analysis

Cells were lysed at 4 °C in ice-cold radioimmunoprecipitation assay buffer (Cell Signaling Technology, 9806), and cell lysates were cleared by brief centrifugation (13,000 × *g*, 15 min). Concentrations of proteins in the supernatant were determined using the BCA assay (Thermo Fisher Scientific, 23225). Before immunoprecipitation, samples containing equal amounts of proteins were precleared with protein A agarose beads (4 °C, 3 h; Santa Cruz Biotechnology, sc-2027) and subsequently incubated with various irrelevant IgG or 5 µg/ml anti-EIF4E antibody (Thermo Fisher Scientific, MA1-089, clone 5D11, 1:200) in the presence of protein A agarose beads for 2 h or overnight at 4 °C with gentle shaking. Following incubation, agarose beads were washed extensively with PBS, and proteins were eluted by boiling in 2× sodium dodecyl sulfate (SDS) sample buffer before SDS–polyacrylamide gel electrophoresis.

### Tandem mass spectrometry

Proteolytic peptides from in-gel trypsin digestion were analyzed using nanoflow reverse-phased liquid chromatography–tandem mass spectrometry (LC-MS/MS). Tryptic peptides were loaded onto a C18 column (PicoChip column packed with 10.5-cm ReproSil C18 [3 µm and 120 Å] chromatography media with the column having an internal diameter of 75 µm and a tip of 15 µm; New Objective Inc). Loading was performed using a Dionex HPLC system (Dionex UltiMate 3000, Thermo Fisher Scientific) operated with a double-split system to provide an in-column nanoflow rate (~300 nl/min). Mobile phases used were 0.1% formic acid for A and 0.1% formic acid in ACN for B. Peptides were eluted off the column using a 52-min gradient (2 to 40% B in 42 min, 40 to 95% B in 1 min, 95% B for 1 min, and 2% B for 8 min) and injected into a linear ion trap MS (LTQ XL, Thermo Fisher Scientific) through electrospray. The LTQ XL was operated in a date-dependent MS/MS mode in which each full MS spectrum (acquired at 30,000 automatic gain control [AGC] targets, 50-ms maximum ion accumulation time, and a precursor ion selection range of mass/charge ratio of 375 to 1800) was followed by MS/MS scans of the eight most abundant molecular ions determined from full MS scan (acquired on the basis of the setting of 1000 signal thresholds, 10,000 AGC targets, 100-ms maximum accumulation time, 2.0-Da isolation width, 30-ms activation time, and 35% normalized collision energy). Dynamic exclusion was enabled to minimize the redundant selection of peptides previously selected for collision-induced dissociation.

### Peptide identification by database search

MS/MS spectra were searched using the Mascot search engine (version 2.4.0, Matrix Science Ltd.) against the UniProt human proteome database (https://www.uniprot.org/proteomes?facets=superkingdom%3AEukaryota&query = %2A). The modifications used were the following: static modification of cysteine (carboxyamidomethylation, +57.05 Da), variable modification of methionine (oxidation, +15.99 Da), and protein N-terminal acetylation. The mass tolerance was set to 1.4 Da for the precursor ions and 0.8 Da for the fragment ions. Peptide identifications were filtered using PeptideProphet and ProteinProphet algorithms with a protein threshold cutoff of 99% and a peptide threshold cutoff of 90% implemented in Scaffold (Proteome Software, Portland, OR, USA).

### Immunofluorescence assay

The cells were fixed with 2% paraformaldehyde and incubated with primary antibodies in PBS with 1% bovine serum albumin overnight at 4 °C, followed by washing and the application of secondary antibodies. After final washing, sections were protected with coverslips with an anti-fading mounting medium sealed with nail polish and stored at 4 °C for preservation. Immunofluorescence images were acquired using a confocal laser scanning microscope (ZEISS LSM 800).

### Biochemical assay

Commercially available assay kits were used to measure the concentrations or activity of iron (Abcam, ab83366), caspase-3 activity (Cell Signaling Technology, 5723), 4HNE (BioVision, E4645-100), HMGB1 (Shino-Test Corporation, 326070442), GSH (Thermo Fisher Scientific, EIAGSHC), ALDH1B1 (Abcam, ab214024), EIF4E (Abcam, ab214564), or NOX1 (NOVUS, NBP2-76746) in the indicated samples according to the manufacturer instructions. Measurement of ALT and BUN in the serum was performed using an IDEXX Catalyst Dx Chemistry Analyzer.

### Luciferase reporter assay

The cap-dependent luciferase reporter plasmid pcDNA-LUC was a gift from Dr. Hsin-Sheng Yang^89^. A dual-luciferase mRNA reporter constructs containing firefly (FF) luciferase, HCV IRES, and renilla (RN) luciferase was a gift from ref. 90. Cells in 12-well plates were transfected with 0.4 μg reporter cDNA and 2 μl Lipofectamine 3000 (Thermo Fisher Scientific, L3000-015). Cell lysates were collected 48 h after transfection and then indicated luciferase activity was measured^44^.

### Affinity pull-down assay

We used MagZ™ Particles System (Promega, V8830) to evaluate the direct binding between the bait protein and the prey protein. In brief, 10 µg His-EIF4E bait protein (NOVUS, NBP1-45314) was added to 30 µl MagZ™ binding particles and incubated for 15 min on a shaker. The MagZ™ particles were washed three times with 200 µl of 20 mM sodium phosphate (pH 7.4) and resuspended with 30 µl of MagZ™ binding/wash buffer. About 5 µl of particles was transferred to new tubes. Particles were resuspended in 175 µl of the MagZ™ binding/wash buffer. About 2 µg ALDH1B1 (OriGene, TP300684) or ALDH3A1 (OriGene, TP302440) protein was added to the prepared bait His-EIF4E/MagZ™ particles and incubated for 60 min on a shaker. The particles were washed three times in the same final wash buffer used in the immobilization, followed by an additional wash of 500 mM imidazole. About 20 µl SDS buffer was added to the particles and incubated for 5 min with shaking, and the samples were collected for SDS-PAGE electrophoresis.

### Iron assay

The relative Fe^2+^ concentration in cells was assessed using an Iron Assay Kit (Sigma-Aldrich, MAK025). Briefly, cells (2 × 10^6^) were homogenized in 4–10 volumes of iron assay buffer and the samples were centrifuged at 16,000 × *g* for 10 min to remove insoluble materials to collect the supernatants. To measure ferrous iron, we added 50 μl samples to sample wells in a 96-well plate and brought the volume to 100 μl per well with 5 μl assay buffer. After incubation of the reaction at 37 °C for 30 min, the absorbance at 593 nm was measured using a microplate reader. The sample concentration was counted based on the standard curve.

### Animal model

We conducted all animal care and experiments in accordance with the Association for Assessment and Accreditation of Laboratory Animal Care guidelines and with approval from our institutional animal care and use committees (Guangzhou Medical University and UT Southwestern Medical Center). The maximal tumor size/burden permitted in the protocol of the institutional animal care and use committees is 2000 mm^3^, and the maximal tumor size/burden was not exceeded in the experiments. All mice were housed under a 12-h light-dark diurnal cycle with controlled temperature (20–25 °C) and relative humidity (40–60%). Food (Laboratory Rodent Diet, LabDiet, 5001) and water were available ad libitum. Experiments were carried out under pathogen-free conditions and the health status of mouse lines was routinely checked by veterinary staff. No wild animals were used in the study. Experiments were carried out with randomly chosen littermates of the same sex and matched by age and body weight. Animals were sacrificed at the indicated time by CO_2_ asphyxia, and blood samples and tissue were collected.

To generate murine subcutaneous tumors, 1.5 × 10^6^ indicated HT-1080 cells in 100 μl PBS were injected subcutaneously into the right of the dorsal midline in 6- to 8-week-old athymic nude female BALB/c mice. Once the tumors reached 60–80 mm^3^ on day 7, mice were randomly allocated into groups and then treated with IKE (40 mg/kg, i.p., once every other day) or at day 7 for 2 weeks. To investigate the role of 4HNE in vivo, athymic nude female mice were injected subcutaneously with HT-1080 cells for 7 days and then given intratumoral treatment with 4HNE (5 mg/kg, once every other day) in the absence or presence of ZVAD-FMK (5 mg/kg, once every other day) or liproxstatin-1 (5 mg/kg, once every other day) at day 7 for 2 weeks. Tumors were measured twice weekly and volumes were calculated using the formula length × width^2^ × π/6.

### Statistical analysis

Data are presented as mean ± SD except where otherwise indicated. Each experiment was repeated in triplicates independently with similar results and representative results are shown. GraphPad Prism (version 8.4.3) was used to collect and analyze data. A one-way (for one independent variable) or two-way (for two independent variables) analysis of variance (ANOVA) with Tukey’s multiple comparisons test was used for comparison among the different groups on all pairwise combinations. No adjustment was made for multiple comparisons. A two-tailed *P* value of <0.05 was considered statistically significant. The exact value of *n* within the figures and replicates is indicated in the figure legends. We did not exclude samples or animals. No statistical methods were used to predetermine sample sizes, but our sample sizes are similar to those generally employed in the field^74^.

### Reporting summary

Further information on research design is available in the Nature Research Reporting Summary linked to this article.

## Supplementary information

Supplementary Information

Description of Additional Supplementary Files

Supplementary Data 1

Supplementary Data 2

Supplementary Data 3

Reporting Summary

## Data Availability

All the data supporting the findings of this study are available within the article and its supplementary information files. Mass spectrometry data generated for this study is available via the MassIVE repository with the following accession number MSV000088802. The sequencing data generated in this study have been deposited at National Center for Biotechnology Information (NCBI) with accession number PRJNA870904. Source data are provided with this paper.

## Source data

Source Data
