# Supplementary material for: A noncanonical function of EIF4E limits ALDH1B1 activity and increases susceptibility to ferroptosis
Source: Nat Commun. 2022 Oct 23;13:6318. doi: 10.1038/s41467-022-34096-w (PMC9588786; doi:10.1038/s41467-022-34096-w)
Supplement: Supplementary file 1 — Supplementary Information [file 41467_2022_34096_MOESM1_ESM.pdf]

Supplemental figures and figure legends

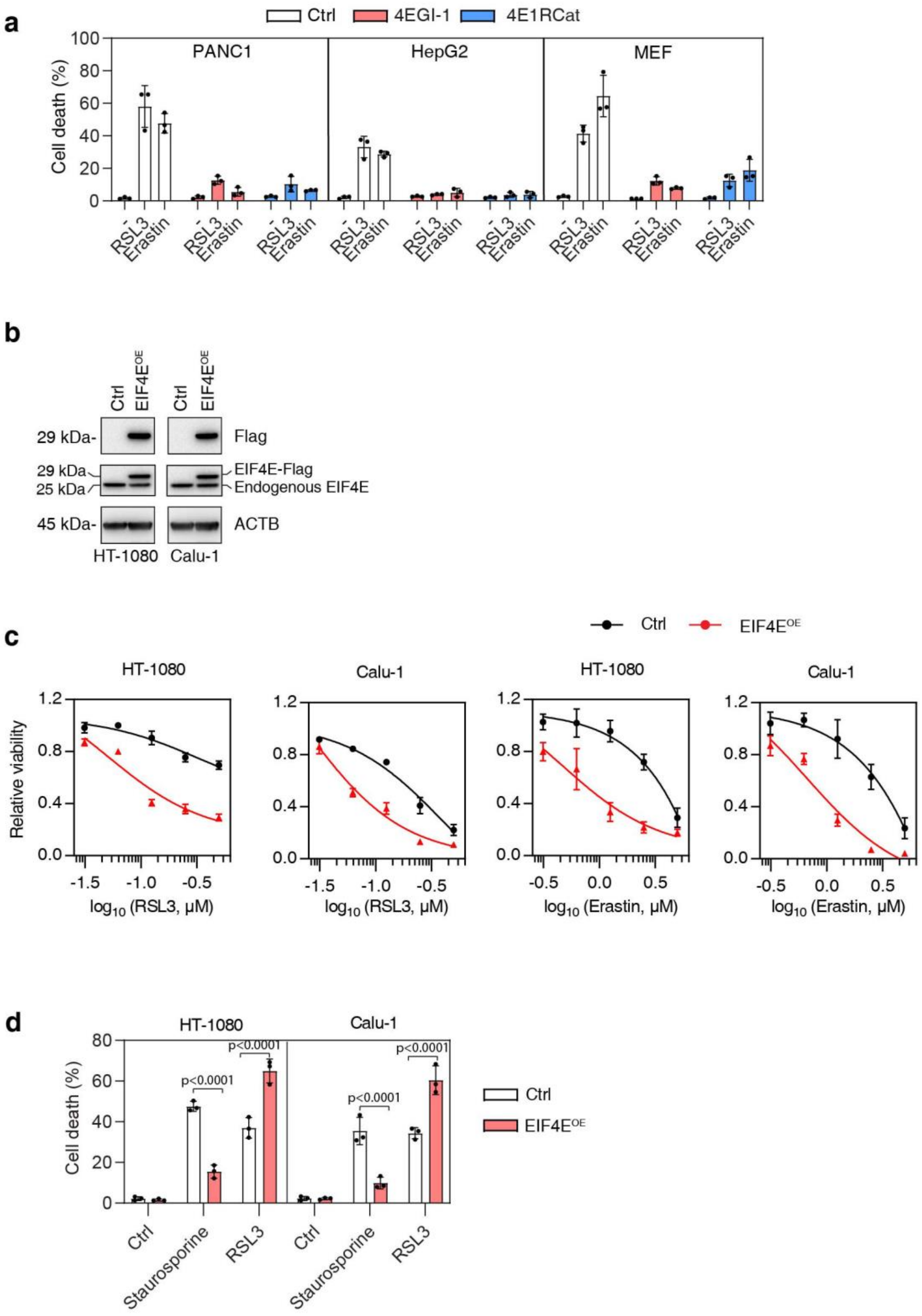

Supplementary Figure 1. Overexpression of EIF4E inhibits activity of RSL3 and erastin

(**a**) Cell death of indicated cells following treatment with RSL3 (PANC1: 0.5 μM; HepG2: 0.5 μM; MEF: 0.5 μM) or erastin (PANC1: 5 μM; HepG2: 10 μM; MEF: 1 μM) in the absence or presence of 4EGI-1 (10 μM) or 4E1RCat (10 μM) for 24 hours (n = 3 biologically independent samples; data are presented as mean ± SD).

(**b**) Western blot analysis of the indicated proteins in control and EIF4E-overexpressing ($EIF4E^{OE}$) HT-1080 and Calu-1 cells.

(**c**) Cell viability of control and EIF4E-overexpressing ($EIF4E^{OE}$) HT-1080 and Calu-1 cells following treatment with RSL3 or erastin at the indicated dose for 24 hours (n = 3 biologically independent samples; data are presented as mean ± SD).

(d) Cell death of indicated cells following treatment with RSL3 (0.5 μM) or staurosporine (0.25 μM) for 24 hours (n = 3 biologically independent samples; two-way ANOVA with Tukey's multiple comparisons test; data are presented as mean ± SD).

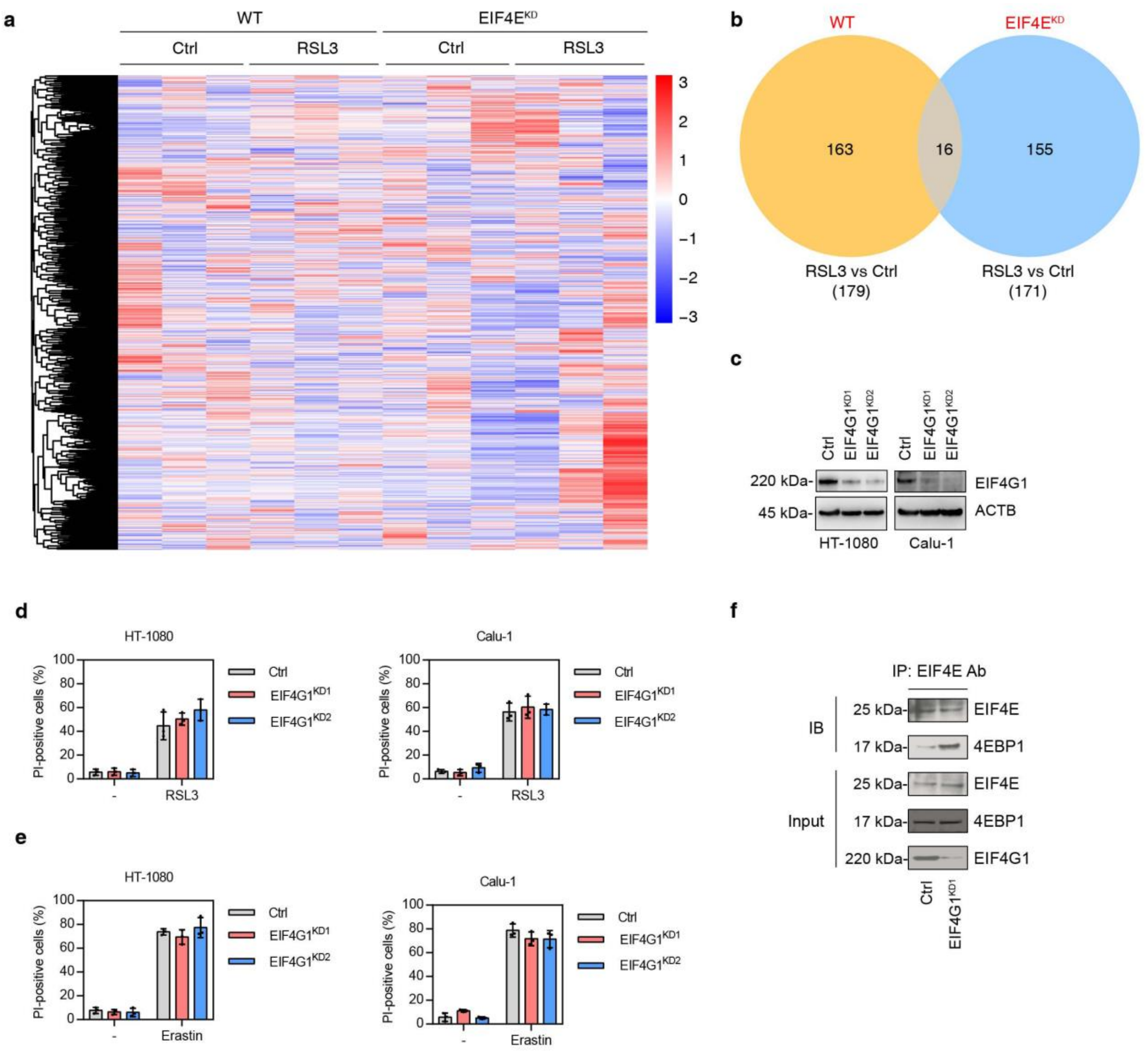

Supplementary Figure 2. EIF4G1 is not required for ferroptosis

(a) Active ribosome profiling with RiboLace. Hierarchical clustering of transcripts altered (>1-fold change) in RSL3 (0.5 µM, 4 h)-treated control and EIF4E knockdown (EIF4E^{KD}) HT-1080 cells (n = 3 biologically independent samples/group).

(b) Venn diagram of differentially expressed mRNA (>1-fold change) in control and EIF4E knockdown (EIF4E^{KD}) cells aftertreatment with RSL3 (0.5 µM, 4 h).

(**c**) Western blot analysis of the indicated proteins in control and EIF4G1 knockdown (EIF4G1^{KD}) HT-1080 and Calu-1 cells.

(**d** and **e**) Propidium iodide (PI) staining analysis of cell death in control and EIF4G1 knockdown ($EIF4G1^{KD}$) HT-1080 and Calu-1 cells following treatment with 0.5 μM RSL3 (B) or 5 μM erastin (C) for 24 hours (n = 3 biologically independent samples; data are presented as mean ± SD).

(**f**) Immunoprecipitation (IP) analysis of EIF4E-binding proteins in control and EIF4G1 knockdown ($EIF4G1^{KD1}$) HT-1080 cells. IB, immunoblot.

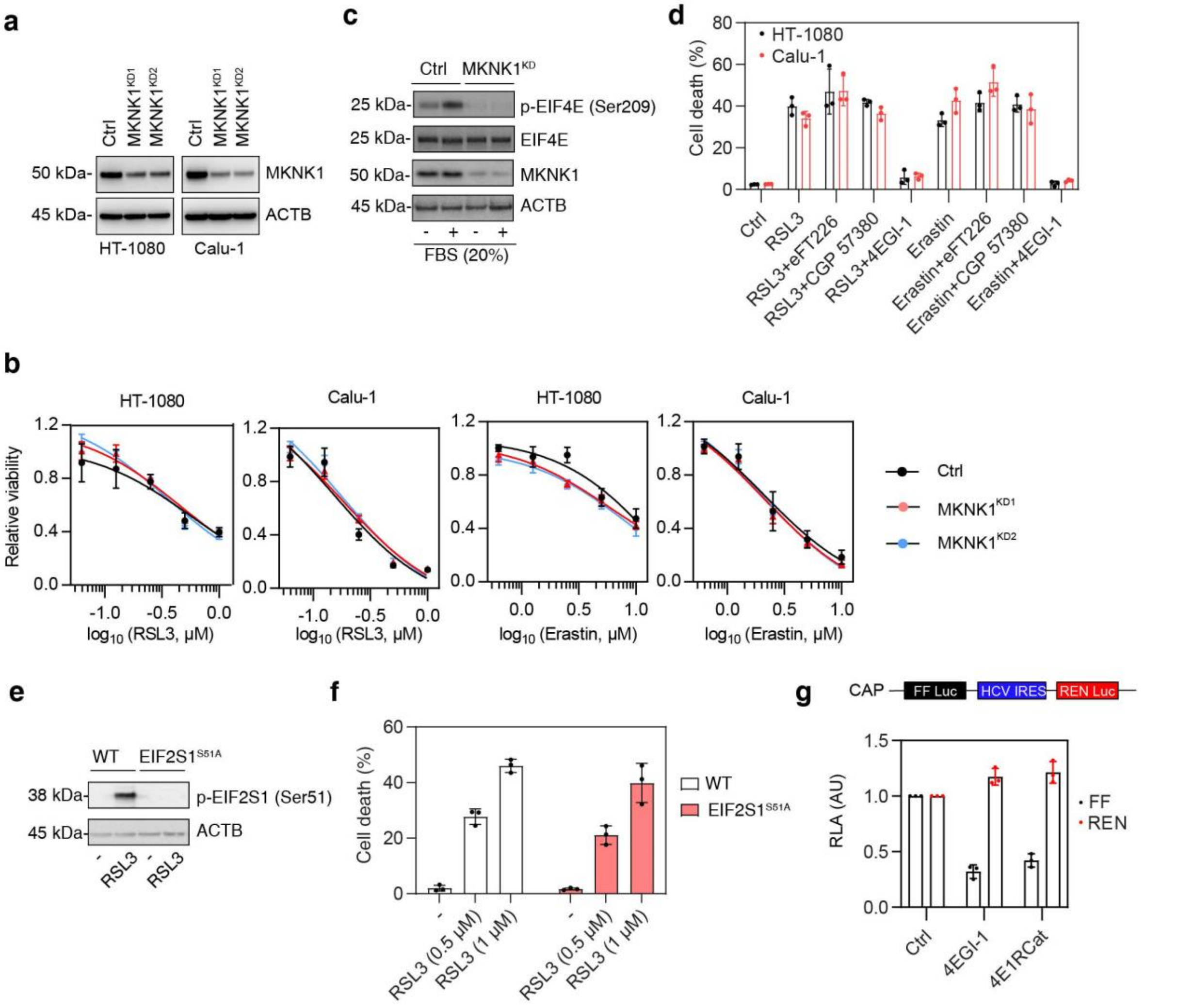

Supplementary Figure 3. MKNK1 and EIF2S1^{S51} is not required for ferroptosis

(**a**) Western blot analysis of the indicated proteins in control and MKNK1 knockdown ($MKNK1^{KD}$) HT-1080 and Calu-1 cells.

(**b**) Cell viability of control and MKNK1 knockdown ($MKNK1^{KD}$) HT-1080 and Calu-1 cells following treatment with RSL3 or erastin at the indicated dose for 24 hours (n = 3 biologically independent samples; data are presented as mean ± SD).

(c) Western blot analysis of the indicated proteins in control and MKNK1 knockdown ($MKNK1^{KD}$) Calu-1 cells following treatment with 20% FBS for 6 hours.

(d) Cell death analysis of HT-1080 and Calu-1 cells following treatment with RSL3 (0.5 µM) or erastin (5 µM) in the absence or presence of eFT226 (50 nM), CGP 57380 (5 µM), or 4EGI-1 (5 µM) for 24 hours (n = 3 biologically independent samples; data are presented as mean ± SD).

(e) Western blot analysis of the indicated proteins in wide type (WT) and EIF2S1^{S51A} U2OS cells following treatment with RSL3 (0.5 μM) for 6 hours.

(f) Cell death analysis of indicated U2OS cells following treatment with RSL3 for 24 hours (n = 3 biologically independent samples; data are presented as mean ± SD).

(g) HT-1080 cells were transfected with a dual luciferase reporter plasmid including HCV IRES activity linked to renilla luciferase (REN) and cap-dependent translation activity linked to firefly luciferase (FF). The transfected cells were treated with 4EGI-1 (10 μM) or 4E1RCat (10 μM) and harvested for luciferase assay (RLA; n = 3 biologically independent samples; data are presented as mean ± SD).

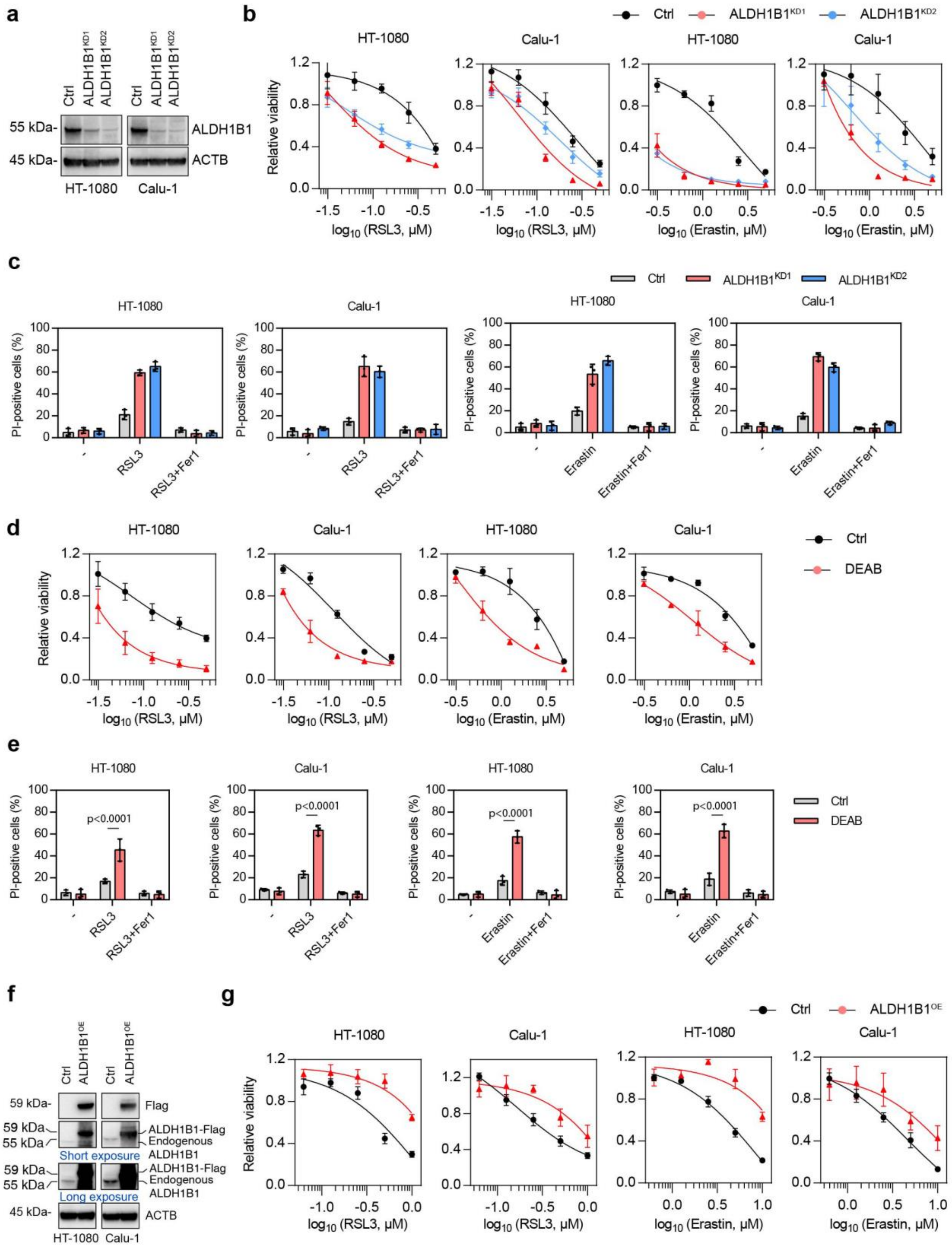

a
Ctrl
ALDH1B1KD1
ALDH1B1KD2
55 kDa-
ALDH1B1
45 kDa-
ACTB
HT-1080
Calu-1
b
Ctrl
ALDH1B1KD1
ALDH1B1KD2
HT-1080
Calu-1
Relative viability
log10 (RSL3, μM)
log10 (Erastin, μM)
c
PI-positive cells (%)
RSL3
RSL3+Fer1
Erastin
Erastin+Fer1
d
DEAB
e
p<0.0001
f
ALDH1B1OE
59 kDa-
Flag
ALDH1B1-Flag
Endogenous ALDH1B1
Short exposure
Long exposure
g

Supplementary Figure 4. ALDH1B1 inhibits ferroptosis

(**a**) Western blot analysis of the indicated proteins in control and ALDH1B1 knockdown ($ALDH1B1^{KD}$) HT-1080 and Calu-1 cells.

(**b**) Cell viability of control and ALDH1B1 knockdown ($ALDH1B1^{KD}$) HT-1080 and Calu-1 cells following treatment with RSL3 or erastin at the indicated dose for 24 hours (n = 3 biologically independent samples; data are presented as mean ± SD).

(**c**) Propidium iodide (PI) staining analysis of cell death in control and ALDH1B1 knockdown ($ALDH1B1^{KD}$) HT-1080 and Calu-1 cells following treatment RSL3 (0.1 μM) or erastin (1 μM) for 24 hours (n = 3 biologically independent samples; data are presented as mean ± SD).

(**d**) Cell viability of HT-1080 and Calu-1 cells following treatment with RSL3 or erastin at the indicated dose in the absence or presence of ALDH inhibitor N, N-diethylaminobenzaldehyde (DEAB; 100 μM) for 24 hours (n = 3 biologically independent samples; data are presented as mean ± SD).

(**e**) Propidium iodide (PI) staining analysis of cell death in HT-1080 and Calu-1 cells following treatment with RSL3 (0.5 μM) or erastin (5 μM) in the absence or presence of ALDH inhibitor N, N-diethylaminobenzaldehyde (DEAB; 100 μM) for 24 hours (n = 3 biologically independent samples; two-way ANOVA with Tukey's multiple comparisons test; data are presented as mean ± SD).

(**f**) Western blot analysis of the indicated proteins in control and ALDH1B1-overexpressing ($ALDH1B1^{OE}$) HT-1080 and Calu-1 cells.

(**g**) Cell viability of control and ALDH1B1-overexpressing ($ALDH1B1^{OE}$) HT-1080 and Calu-1 cells following treatment with RSL3 or erastin at the indicated dose for 24 hours (n = 3 biologically independent samples; data are presented as mean ± SD).

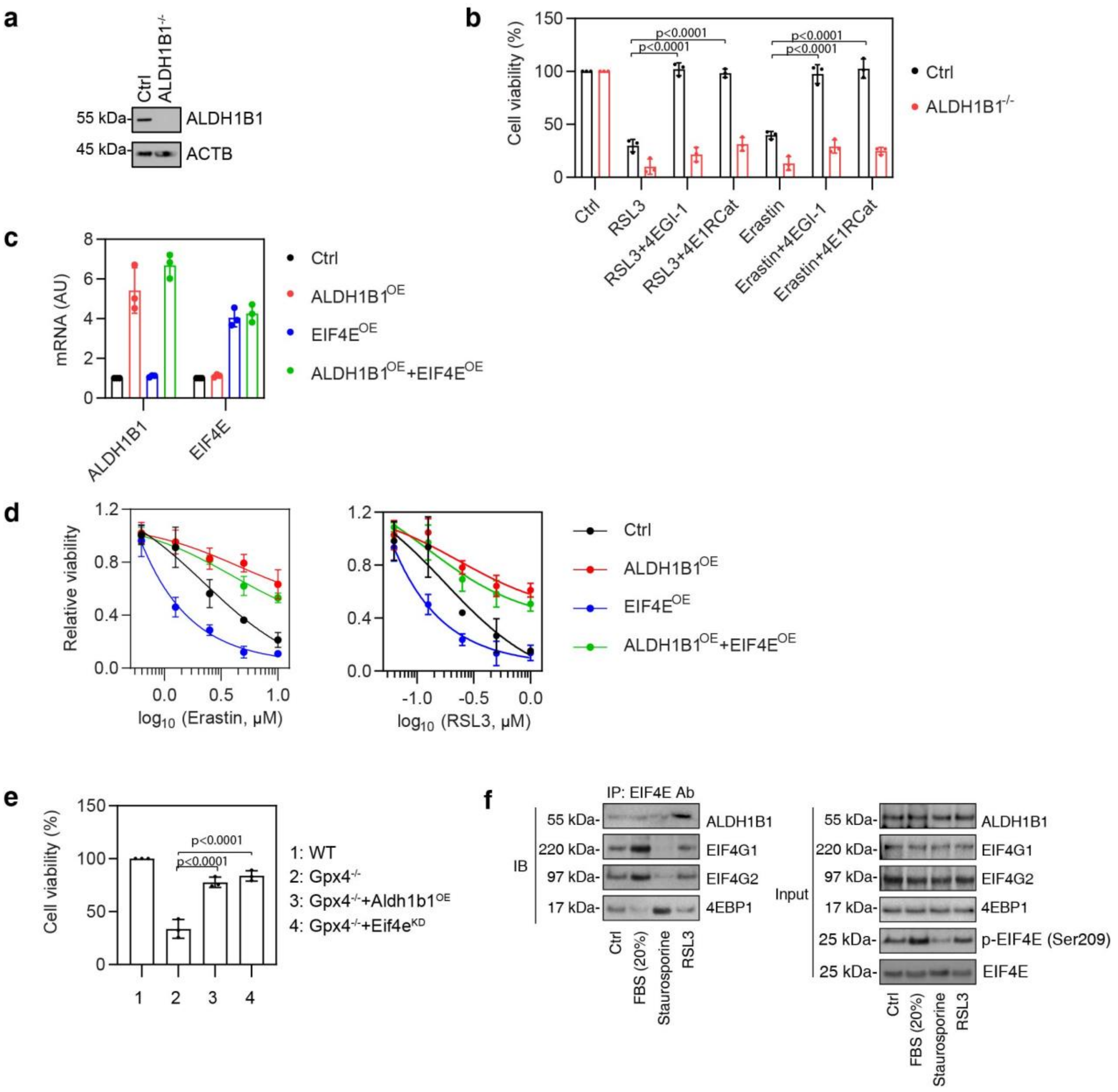

Supplementary Figure 5. ALDH1B1 inhibits ferroptosis in wild type and EIF4E-overexpressed cells.

(**a**) Western blot analysis of ALDH1B1 expression in CRISPR/Cas9-mediated knockout of the ALDH1B1 gene in Calu-1 cells.

(**b**) Cell viability of indicated Calu-1 cells following treatment with RSL3 (0.25 μM), erastin (2.5 μM) in the absence or presence of 4EGI-1 (10 μM) or 4E1RCat (10 μM) for 24 hours (n = 3 biologically independent samples; two-way ANOVA with Tukey's multiple comparisons test; data are presented as mean ± SD).

(**c**) qPCR analysis of ALDH1B1 and ELF4E expression in indicated Calu-1 cells (n = 3 biologically independent samples; data are presented as mean ± SD).

(**d**) Cell viability of indicated Calu-1 cells following treatment with RSL3 or erastin at the indicated dose for 24 hours (n = 3 biologically independent samples; data are presented as mean ± SD).

(**e**) Cell viability assay of indicated Pfa1 cells after knockout of Gpx4 for 24 hours (n = 3 biologically independent samples; data are presented as mean ± SD).

(**f**) Immunoprecipitation (IP) analysis of EIF4E-binding proteins in HT-1080 cells following treatment with RSL3 (0.5 μM), 20%FBS, or staurosporine (0.25 μM) for 4 hours. IB, immunoblot.

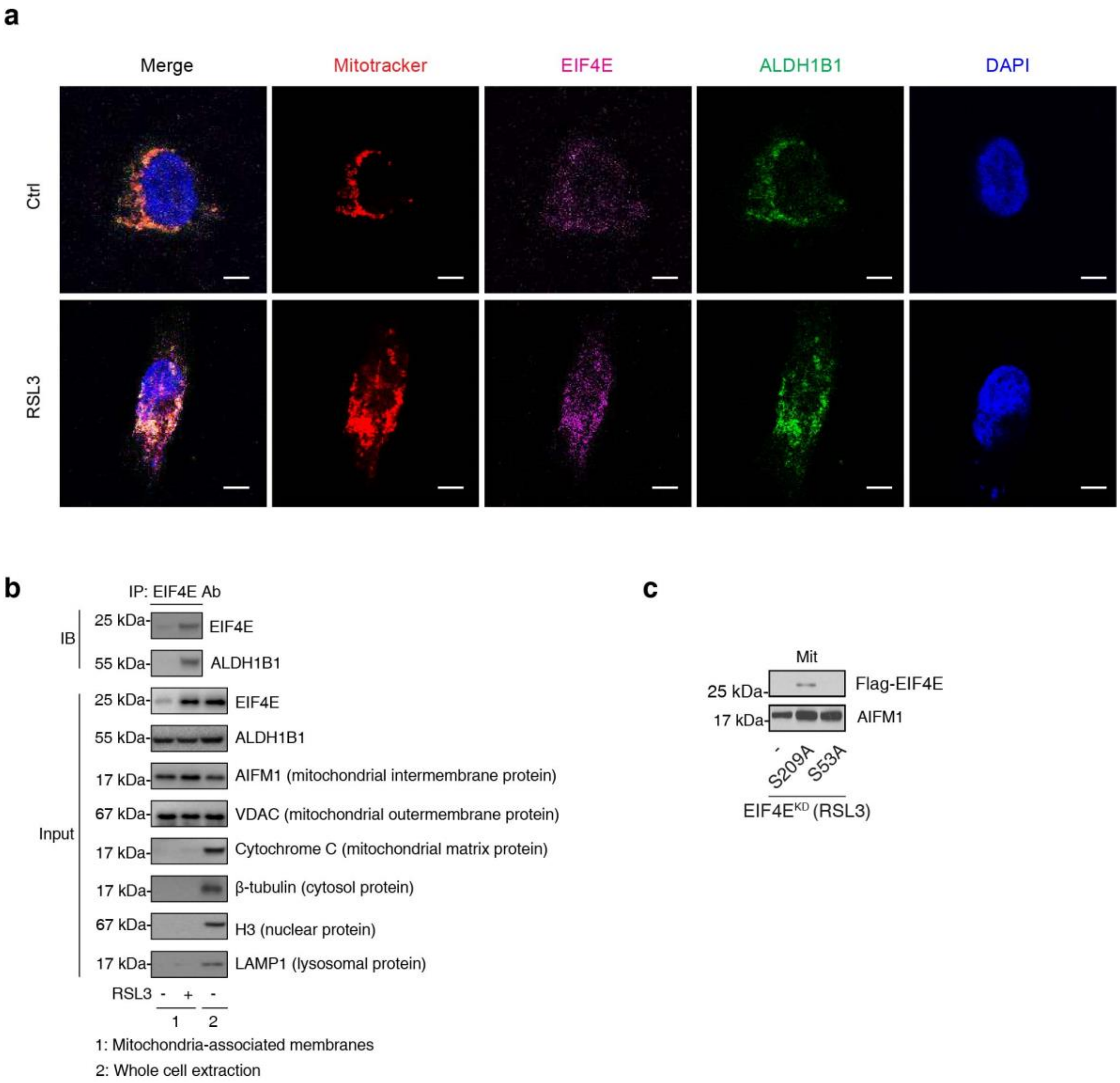

Supplementary Figure 6. Colocalization between ALDH1B1 and EIF4E in mitochondria.

(a) Representative immunofluorescence images showing staining of ALDH1B1 (green), EIF4E (purple), and mitochondria (red) in HT-1080 cells following treatment with RSL3 (0.5 μM) for 4 hours (scale bar = 10 μm).

(b) Immunoprecipitation (IP) analysis of the EIF4E-ALDH1B1 complex in mitochondria-associated membranes of HT-1080 cells following treatment with RSL3 (0.5 μM) for 4 hours.

(c) Western blot analysis of EIF4E expression in mitochondria of the indicated HT-1080 cells after treatment with RSL3 (0.5 μM) for 4 hours.

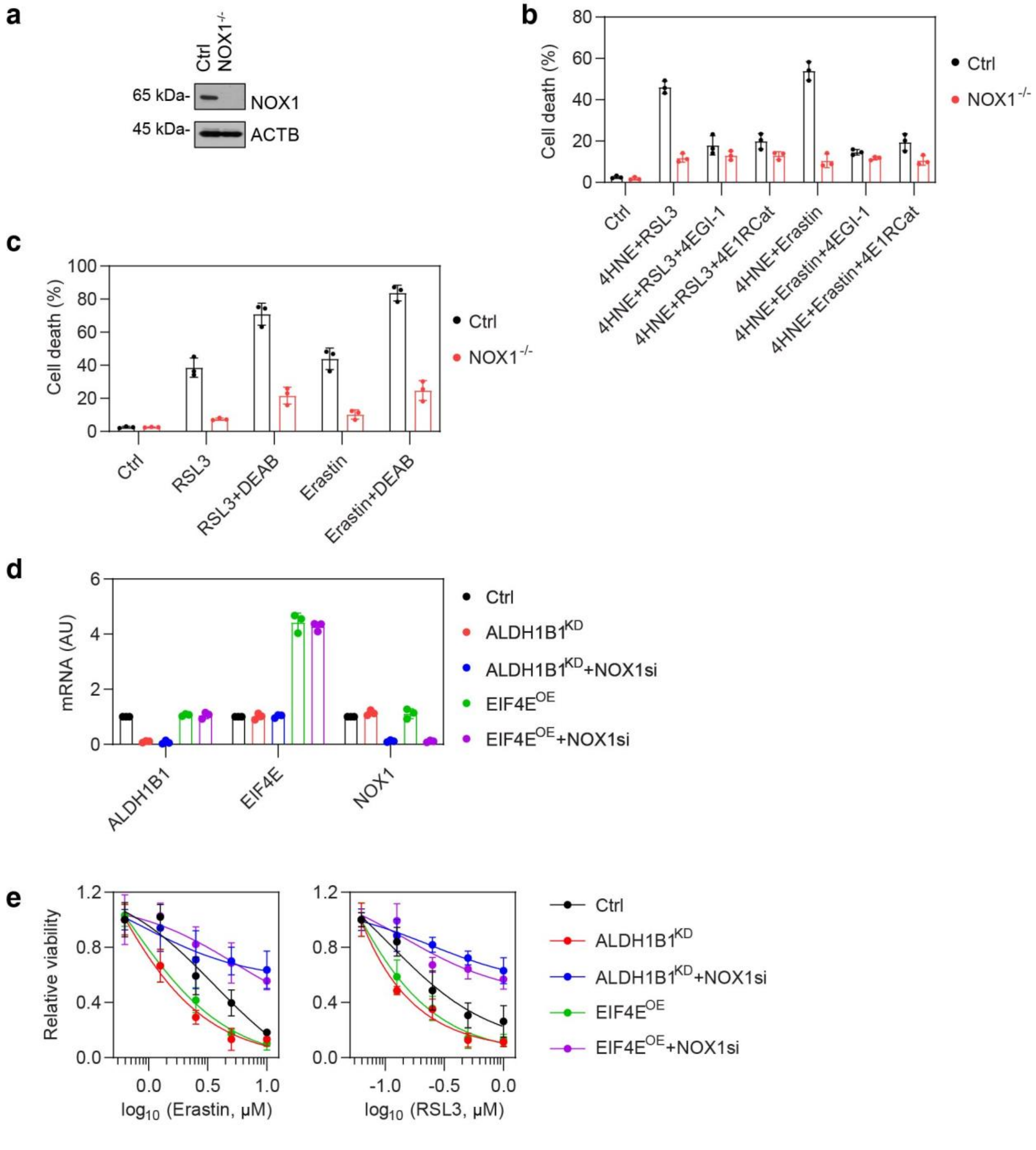

Supplementary Figure 7. NOX1 mediates ferroptosis in EIF4E-overexpressed or ALDH1B1-knockdown Calu-1 cells.

(**a**) Western blot analysis of NOX1 expression in CRISPR/Cas9-mediated knockout of the NOX1 gene in Calu-1 cells.

(**b**) Cell death of indicated Calu-1 cells following treatment with 4HNE (12.5 μM) in the presence of RSL3 (0.1 μM), erastin (1 μM), 4EGI-1 (10 μM) or 4E1RCat (10 μM) for 24 hours (n = 3 biologically independent samples; data are presented as mean ± SD).
(c) Cell death of indicated Calu-1 cells following treatment with RSL3 (0.25 μM), erastin (2.5 μM) in the absence or presence of DEAB (100 μM) for 24 hours (n = 3 biologically independent samples; data are presented as mean ± SD).
(**d**) qPCR analysis of ALDH1B1, ELF4E, and NOX1 expression in indicated Calu-1 cells (n = 3 biologically independent samples; data are presented as mean ± SD).
(**E**) Cell viability of indicated Calu-1 cells following treatment with RSL3 or erastin at the indicated dose for 24 hours (n = 3 biologically independent samples; data are presented as mean ± SD).

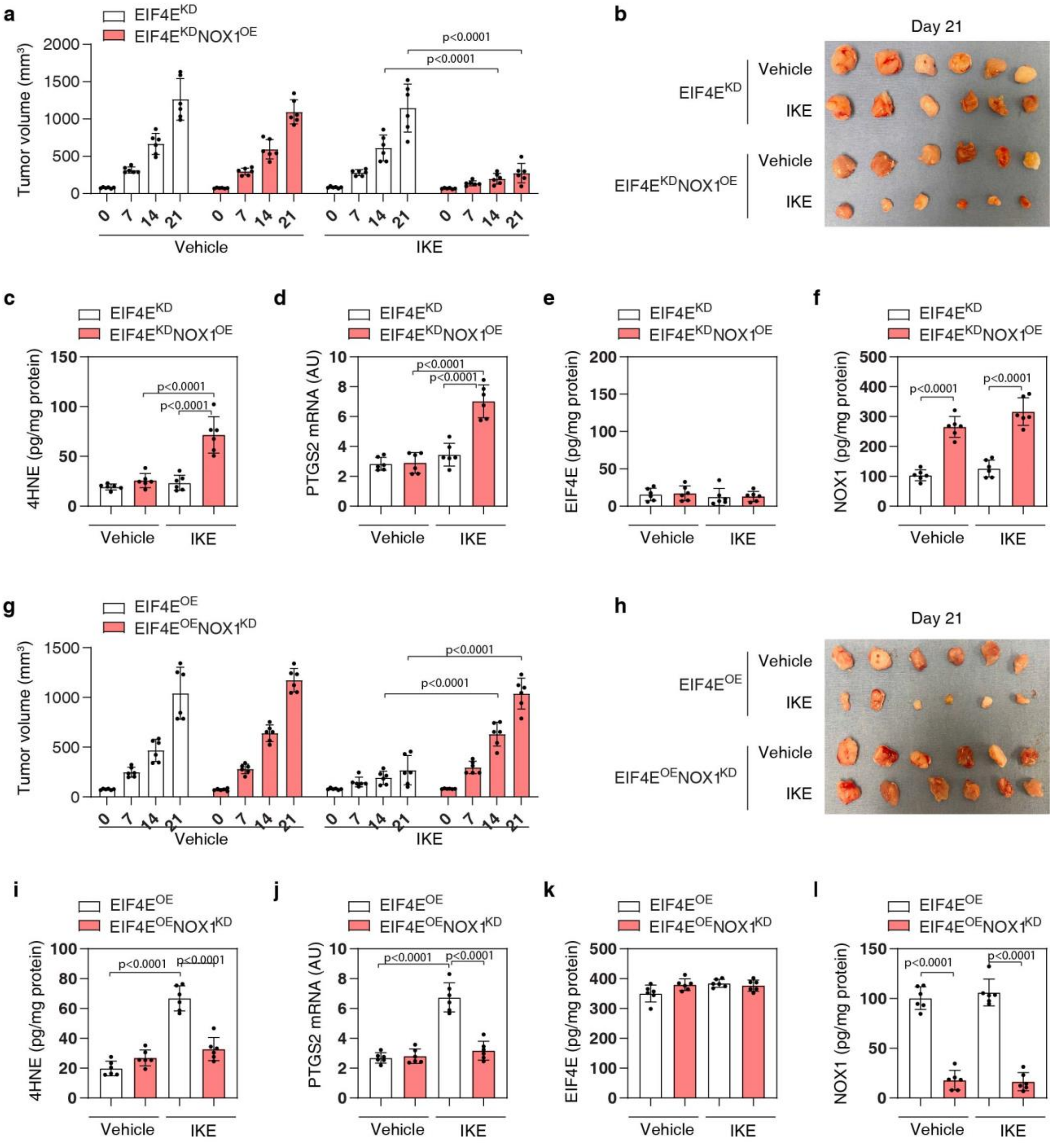

Supplementary Figure 8. NOX1 mediates EIF4E-dependent ferroptosis *in vivo*.

(a) Athymic nude mice were injected subcutaneously with indicated HT-1080 cells for 7 days and then treated with IKE (40 mg/kg, i.p., once every other day) at day 7 for 2 weeks. Tumor volumes were calculated weekly (n = 6 mice/group; two-way ANOVA with Tukey's multiple comparisons test; data are presented as mean ± SD).

(b) Photographs of isolated tumors at day 14 after treatment.

(c-f) The levels of 4HNE (c), PTGS2 mRNA (d), EIF4E protein (e), and NOX1 protein (f) in isolated tumors at day 14 after treatment were assayed (n = 6 mice/group; two-way ANOVA with Tukey's multiple comparisons test; data are presented as mean ± SD).

(g) Athymic nude mice were injected subcutaneously with indicated HT-1080 cells for 7 days and then treated with IKE (40 mg/kg, i.p., once every other day) at day 7 for 2 weeks. Tumor volumes were calculated weekly (n = 6 mice/group; two-way ANOVA with Tukey's multiple comparisons test; data are presented as mean ± SD).

(h) Photographs of isolated tumors at day 14 after treatment.

(i-l) The levels of 4HNE (i), PTGS2 mRNA (j), EIF4E protein (k), and NOX1 protein (l) in isolated tumors at day 14 after treatment were assayed (n = 6 mice/group; two-way ANOVA with Tukey's multiple comparisons test; data are presented as mean ± SD).

Supplementary Table 1. Reagent sources

REAGENT or RESOURCE	SOURCE	IDENTIFIER
**Antibodies**		
EIF4E	Cell Signaling Technology	9742
EIF4E	Thermo Fisher Scientific	MA1-089
ACTB/Actin	Cell Signaling Technology	3700
Flag	Cell Signaling Technology	8146
EIF4G1	Cell Signaling Technology	2469
EIF4G2	Cell Signaling Technology	2182
Phospho-EIF2S1 (Ser51)	Cell Signaling Technology	3398
MKNK1/MNK1	Cell Signaling Technology	2195
Phospho-EIF4E (Ser209)	Cell Signaling Technology	9741
MAP1LC3	Cell Signaling Technology	12741
AIFM1/AIF	Cell Signaling Technology	5318
Histone H3	Cell Signaling Technology	9715
SOD1	Abcam	ab16831
4HNE	Abcam	ab46545
ALDH1B1	Proteintech	15560-1-AP
ALDH3A1	Abcam	ab129022
ALDH3A2	Thermo Fisher Scientific	15090-1-AP
NOX1	NOVUS	NBP1-31546
CPNE3	Thermo Fisher Scientific	PA5-56186
PTGES2	Santa Cruz Biotechnology	sc-514224
IARS	Abcam	ab151557
GSTO1	NOVUS	NBP1-33763
HSD17B4	Abcam	ab97971
CEPT1	Santa Cruz Biotechnology	sc-133421
VDAC	Cell Signaling Technology	4866
Cytochrome C	Cell Signaling Technology	4280
LAMP1	Cell Signaling Technology	9091
β-tubulin	Cell Signaling Technology	2128

**Chemicals, Peptides, and Recombinant Proteins**		
4EGI-1	Selleck Chemicals	S7369
4E1RCat	Selleck Chemicals	S7370
Erastin	Selleck Chemicals	S7242
RSL3	Selleck Chemicals	S8155
FIN56	Selleck Chemicals	S8254
Ferrostatin-1	Selleck Chemicals	S7243
Liproxstatin-1	Selleck Chemicals	S7699
Necrosulfonamide	Selleck Chemicals	S8251
ZVAD-FMK	Selleck Chemicals	S7023
Deferoxamine	Selleck Chemicals	S5685
Diphenyleneiodonium	Selleck Chemicals	S8639
Staurosporine	Selleck Chemicals	S1421
eFT226	MedChemExpress	HY-112163
CGP 57380	Selleck Chemicals	S7421
ML162	Cayman Chemical	20455
ML210	Cayman Chemical	23282
FINO2	Cayman Chemical	25096
4HNE	Cayman Chemical	32100
4-diethylaminobenzaldehyde	Sigma-Aldrich	D86256
2-acetylphenothiazine	Sigma-Aldrich	175226
DPPH	Sigma-Aldrich	D9132
3-(2-pyridyl)-5,6-diphenyl-1,2,4-triazine-p,p′-disulfonic acid monosodium salt hydrate (FerroZine Iron Reagent)	Sigma-Aldrich	160601
AAPH	Sigma-Aldrich	440914
STY-BODIPY	Cayman Chemical	27089
Egg phosphatidylcholine	Encapsula NanoSciences	CPC-602
IL4	BioLegend	574002
EIF4E	NOVUS	NBP1-45314
ALDH1B1	OriGene	TP300684

ALDH3A1	OriGene	TP302440
Iron (II) chloride	Sigma-Aldrich	372870
Protease inhibitor cocktail	Sigma-Aldrich	P8340
Puromycin	Sigma-Aldrich	MABE343
Hoechst 33342	Thermo Fisher Scientific	62249
SuperSignal West Pico chemiluminescent substrate	Thermo Fisher Scientific	34080
SuperSignal West Femto maximum sensitivity substrate	Thermo Fisher Scientific	34095
PBS	Thermo Fisher Scientific	AM9625
Lipofectamine 3000	Thermo Fisher Scientific	L3000-015
Cell lysis buffer	Cell Signaling Technology	9803
Puromycin	InvivoGen	ant-pr-1
MitoTracker	Invitrogen	M7512
4%-12% Criterion XT Bis-Tris gels	Bio-Rad	3450124
PVDF membranes	Bio-Rad	1620233
XT MES running buffer	Bio-Rad	1610789
**Critical Commercial Assays**		
Cell Counting Kit-8 (CCK8) solutions	Bimake	B34304
BCA Assay Kit	Thermo Fisher Scientific	23225
Propidium iodide solutions	Thermo Fisher Scientific	R37108
BODIPY 581/591 C11	Thermo Fisher Scientific	D3861
Iron assay kit	Abcam	ab83366
HMGB1 ELISA kit	Sino-Test Corporation	326070442
ALDH1B1 ELISA kit	Abcam	ab214024
EIF4E ELISA kit	Abcam	ab214564
NOX1 ELISA kit	NOVUS	NBP2-76746
4HNE ELISA Kit	BioVision	E4645-100
Caspase-3 activity assay kit	Cell Signaling Technology	5723
GSH assay kit	Thermo Fisher Scientific	EIAGSHC
Iron assay kit	Sigma-Aldrich	MAK025
Cell fractionation kit	Cell Signaling Technology	9038

**Experimental Models: Cell Lines**		
HT-1080	ATCC	CCL-121
Calu-1	ATCC	HTB-54
PANC1	ATCC	CRL-1469
HepG2	ATCC	HB-8065
MEF	ATCC	SCRC-1008
Gpx4$^{-/-}$ Pfa1	Marcus Conrad	[46]
293FT	Thermo Fisher Scientific	R70007
**Experimental Models: Organisms/Strains**		
Athymic nude	Charles River	490
**Oligonucleotides**		
Human *EIF4E* shRNA (1) (Sequence: CCGGCCACTCTGTAATAGTTCAGTACTCGA GTACTGAACTATTACAGAGTGGTTTTTG)	Sigma-Aldrich	This paper
Human *EIF4E* shRNA (2) (Sequence: CCGGCGGCTGATCTCCAAGTTTGATCTCGA GATCAAACTTGGAGATCAGCCGTTTTTG)	Sigma-Aldrich	This paper
Human *EIF4G1* shRNA (1) (Sequence: CCGGGCCCTTGTAGTGACCTTAGAACTCGA GTTCTAAGGTCACTACAAGGGCTTTTTG)	Sigma-Aldrich	This paper
Human *EIF4G1* shRNA (2) (Sequence: CCGGGCAGATAGTATCCAACACGTTCTCGA GAACGTGTTGGATACTATCTGCTTTTTG)	Sigma-Aldrich	This paper
Human *ALDH1B1* shRNA (1) (Sequence: CCGGTCGAGAGAACCGTGGAGAAAGCTCG AGCTTTCTCCACGGTTCTCTCGATTTTTG)	Sigma-Aldrich	This paper
Human *ALDH1B1* shRNA (2)	Sigma-Aldrich	This paper

(Sequence: CCGGGAATCCATCTACAATGAGTTTCTCGAGAAACTCATTGTAGATGGATTCTTTTT)		
Human *MKNK1* shRNA (1) (Sequence: CCGGCCTATGCCAAAGTTCAAGGTGCTCGAGCACCTTGAACTTTGGCATAGGTTTTTG)	Sigma-Aldrich	This paper
Human *MKNK1* shRNA (2) (Sequence: CCGGCCTATGCCAAAGTTCAAGGTGCTCGAGCACCTTGAACTTTGGCATAGGTTTTTTG)	Sigma-Aldrich	This paper
Human *CPNE3* siRNA	Sigma-Aldrich	SASI_Hs01_00225474
Human *PTGES2* siRNA	Sigma-Aldrich	SASI_Hs01_00046708
Human *IARS* siRNA	Sigma-Aldrich	SASI_Hs01_00108173
Human *ALDH1B1* siRNA	Sigma-Aldrich	SASI_Hs01_00186297
Human *GSTO1* siRNA	Sigma-Aldrich	SASI_Hs01_00110859
Human *HSD17B4* siRNA	Sigma-Aldrich	SASI_Hs01_00046318
Human *CEPT1* siRNA	Sigma-Aldrich	SASI_Hs01_00205501
Human *NOX1* siRNA	Sigma-Aldrich	SASI_Hs02_00342845
Human *CYBB/NOX2* siRNA	Sigma-Aldrich	SASI_Hs01_00086110
Mouse *Eif4e* siRNA	Sigma-Aldrich	SASI_Mm01_00070393
Human *ALDH1B1* gRNA	Sigma-Aldrich	HSPD0000001482

Human *NOX1* gRNA	Sigma-Aldrich	HSPD0000079614
Human *EIF4E* cDNA (with Flag)	OriGene	RC207333
Human *ALDH1B1* cDNA (with Flag)	OriGene	RC200684
Mouse *Aldh1b1* cDNA (without Flag)	OriGene	MC203989
Human *NOX1* cDNA (without Flag)	OriGene	SC123944
EIF4E S209A (with Flag)	Xuedong Fang	[90]
EIF4E S53A (with Flag)	Yan Zhang	[52]
pcDNA-LUC	Hsin-Sheng Yang	[88]
Human *RNA18S RNA* primers: 5'-CTACCACATCCAAGGAAGCA-3' and 5'-TTTTTCGTCACTACCTCCCCG-3'	Sigma-Aldrich	This paper
Human *CPNE3* primers:<br>F: GTTTTGGCGCTCAGATACCTCC<br>R: GACAAGACCGATACGCCTCTAC	Sigma-Aldrich	This paper
Human *PTGES2* primers:<br>F: CCTCTATGAGGCTGCTGACAAG<br>R: ATCACACGCAGCACGCCATACA	Sigma-Aldrich	This paper
Human *IARS* primers:<br>F: CCTCTTTGGACAACCGCCTTTC<br>R: GGCATCAGCACCATACTTCTGG	Sigma-Aldrich	This paper
Human *ALDH1B1* primers:<br>F: CAGGTGGACAAGGAGCAGTTTG<br>R: ACGCCACCAAAGACAGTAGGCT	Sigma-Aldrich	This paper
Human *GSTO1* primers:<br>F: GAAGACGACCTTCTTTGGTGGC<br>R: CTTCATGGCTGCCATCCACAGT	Sigma-Aldrich	This paper
Human *HSD17B4* primers:<br>F: GAGAATGCCAGCAAGCCTCAGA<br>R: GCTGTAGACGTTGCACGACTAG	Sigma-Aldrich	This paper
Human *CEPT1* primers:<br>F: TTGCTGGCAGTGATTGGAGGAC<br>R: CACCACCTGTGAAGATTACACGG	Sigma-Aldrich	This paper

Human *NOX1* primers:<br>F: GGTTTTACCGCTCCCAGCAGAA<br>R: CTTCCATGCTGAAGCCACGCTT	Sigma-Aldrich	This paper
Human *CYBB/NOX2* primers:<br>F: CTCTGAACTTGGAGACAGGCAAA<br>R: CACAGCGTGATGACAACTCCAG	Sigma-Aldrich	This paper
Human *SLC7A11* primers:<br>F: TCCTGCTTTGGCTCCATGAACG<br>R: AGAGGAGTGTGCTTGCGGACAT	Sigma-Aldrich	This paper
Human *GPX4* primers:<br>F: ACAAGAACGGCTGCGTGGTGAA<br>R: GCCACACACTTGTGGAGCTAGA	Sigma-Aldrich	This paper
Human *ACSL4* primers:<br>F: GCTATCTCCTCAGACACACCGA<br>R: AGGTGCTCCAACTCTGCCAGTA	Sigma-Aldrich	This paper
Human *POR* primers:<br>F: ACTCTGCTCTCGTCAACCAGCT<br>R: TGGGTGCTTCTTGTTGGACTCC	Sigma-Aldrich	This paper
Human *ALDH1B1* primers:<br>F: CAGGTGGACAAGGAGCAGTTTG<br>R: ACGCCACCAAAGACAGTAGGCT	Sigma-Aldrich	This paper
Human *EIF4E* primers:<br>F: ATGCCTGGCTGTGACTACTCAC<br>R: GAGGTCACTTCGTCTCTGCTGT	Sigma-Aldrich	This paper
Mouse *Ptgs2* primers:<br>F: GCGACATACTCAAGCAGGAGCA<br>R: AGTGGTAACCGCTCAGGTGTTG	Sigma-Aldrich	This paper
**Software and Algorithms**		
Image Lab software	Bio-Rad	http://www.biorad.com/enus/product/imagelabsoftware?ID=KRE6P5E8Z

CFX Manager software 2.0	Bio-Rad	http://www.biorad.com/enus/sku/1845000-cfx-managersoftware?ID=1845000
GraphPad Prism 8.4.3	GraphPad	https://www.graphpad.com/scientific-software/prism/
Image J 1.52v	NIH	https://imagej.nih.gov/ij/notes.html
Mascot search engine 2.4.0	Matrix Science Ltd.	https://www.matrixscience.com/
