# Supplementary material for: A noncanonical function of EIF4E limits ALDH1B1 activity and increases susceptibility to ferroptosis
Source: Nat Commun. 2022 Oct 23;13:6318. doi: 10.1038/s41467-022-34096-w (PMC9588786; doi:10.1038/s41467-022-34096-w)
Supplement: Supplementary file 2 — Description of Additional Supplementary Files [file 41467_2022_34096_MOESM2_ESM.pdf]

Description of Additional Supplementary Files

File name: Supplementary Data 1

Description: Cell viability of HT-1080 cells treated with the GPX4 inhibitor RSL3 in the absence or presence of a panel of 431 target-selective inhibitors (all used at 10 μM).

File name: Supplementary Data 2

Description: Translatome of RSL3-treated wild type (WT) or EIF4E-knockdown HT-1080 cells.

File name: Supplementary Data 3

Description: The interactome of EIF4E during RSL3-induced ferroptosis in HT-1080 and Calu-1 cells using immunoprecipitation in combination with mass spectrometry.
