# Supplementary material for: A noncanonical function of EIF4E limits ALDH1B1 activity and increases susceptibility to ferroptosis
Source: Nat Commun. 2022 Oct 23;13:6318. doi: 10.1038/s41467-022-34096-w (PMC9588786; doi:10.1038/s41467-022-34096-w)
Supplement: Supplementary file 6 — Reporting Summary [file 41467_2022_34096_MOESM6_ESM.pdf]

nature portfolio

Corresponding author(s): Xin Chen, Daolin Tang, Rui Kang, Jinbao Liu

Last updated by author(s): Oct 10, 2022

Reporting Summary

Nature Portfolio wishes to improve the reproducibility of the work that we publish. This form provides structure for consistency and transparency in reporting. For further information on Nature Portfolio policies, see our Editorial Policies and the Editorial Policy Checklist.

Statistics

For all statistical analyses, confirm that the following items are present in the figure legend, table legend, main text, or Methods section.

n/a	Confirmed	
☐	☒	The exact sample size (*n*) for each experimental group/condition, given as a discrete number and unit of measurement
☐	☒	A statement on whether measurements were taken from distinct samples or whether the same sample was measured repeatedly
☐	☒	The statistical test(s) used AND whether they are one- or two-sided<br>*Only common tests should be described solely by name; describe more complex techniques in the Methods section.*
☒	☐	A description of all covariates tested
☒	☐	A description of any assumptions or corrections, such as tests of normality and adjustment for multiple comparisons
☐	☒	A full description of the statistical parameters including central tendency (e.g. means) or other basic estimates (e.g. regression coefficient) AND variation (e.g. standard deviation) or associated estimates of uncertainty (e.g. confidence intervals)
☐	☒	For null hypothesis testing, the test statistic (e.g. *F*, *t*, *r*) with confidence intervals, effect sizes, degrees of freedom and *P* value noted<br>*Give P values as exact values whenever suitable.*
☒	☐	For Bayesian analysis, information on the choice of priors and Markov chain Monte Carlo settings
☒	☐	For hierarchical and complex designs, identification of the appropriate level for tests and full reporting of outcomes
☒	☐	Estimates of effect sizes (e.g. Cohen's *d*, Pearson's *r*), indicating how they were calculated

Our web collection on statistics for biologists contains articles on many of the points above.

Software and code

Policy information about availability of computer code

Data collection	GraphPad Prism 8.4.3 was used to collect and analyze data.
Data analysis	GraphPad Prism 8.4.3 was used to collect and analyze data. Q-PCR analysis was performed using Bio-Rad CFX Manager software 3.1. Image analysis of lipid peroxidation assay or Western blot was conducted with Image J software (version 1.52v). Western blots were analyzed using Image Lab Software (Bio-Rad, version 6.1).

For manuscripts utilizing custom algorithms or software that are central to the research but not yet described in published literature, software must be made available to editors and reviewers. We strongly encourage code deposition in a community repository (e.g. GitHub). See the Nature Portfolio guidelines for submitting code & software for further information.

Data

Policy information about availability of data

All manuscripts must include a data availability statement. This statement should provide the following information, where applicable:

- Accession codes, unique identifiers, or web links for publicly available datasets
- A description of any restrictions on data availability
- For clinical datasets or third party data, please ensure that the statement adheres to our policy

All the other data supporting the findings of this study are available within the article and its supplementary information files. MS/MS spectra were searched using the UniProt human proteome database (https://www.uniprot.org/proteomes?facets=superkingdom%3AEukaryota&query=%2A).Mass spectrometry data generated

for this study is available via the MassIVE repository with the following accession number MSV000088802 (https://massive.ucsd.edu/ProteoSAFe/dataset.jsp?task=f9e01655bcfa49d0af5ccea73927a58c). The sequencing data generated in this study have been deposited at National Center for Biotechnology Information (NCBI) with accession number PRJNA870904 (https://www.ncbi.nlm.nih.gov/bioproject/PRJNA870904). Source data are provided with this paper.

Human research participants

Policy information about studies involving human research participants and Sex and Gender in Research.

Reporting on sex and gender	No human research involved.
Population characteristics	No human research involved.
Recruitment	No human research involved.
Ethics oversight	No human research involved.

Note that full information on the approval of the study protocol must also be provided in the manuscript.

Field-specific reporting

Please select the one below that is the best fit for your research. If you are not sure, read the appropriate sections before making your selection.

[X] Life sciences [ ] Behavioural & social sciences [ ] Ecological, evolutionary & environmental sciences

For a reference copy of the document with all sections, see nature.com/documents/nr-reporting-summary-flat.pdf

Life sciences study design

All studies must disclose on these points even when the disclosure is negative.

Sample size	No statistical methods were used to predetermine sample sizes. Generally accepted sample sizes were used, with reproducible differences between conditions indicating that the chosen sample sizes were sufficient.
Data exclusions	no data were excluded
Replication	Replicates in all assays were confirmatory and the extent described within the text and shown in the main figures.
Randomization	Animal experiments were carried out with randomly chosen littermates of the same sex and matched by age and body weight. For in vitrostudies, all cells from multiple dishes were combined and then plated into wells that were treated with various drugs. Thus, all treatmentgroups came from the same cell stock. Biological independent experiments were performed on independent aliquots of cells thawed from theliquid nitrogen freezer.
Blinding	Animal treatments were performed by technicians who were not blind, but not involved in sample measurement. All in vitro experimentswere not blind, because postdoctoral fellows had their own independent projects, and it is impossible for others to replace them to treat cellsand analyze samples.

Reporting for specific materials, systems and methods

We require information from authors about some types of materials, experimental systems and methods used in many studies. Here, indicate whether each material, system or method listed is relevant to your study. If you are not sure if a list item applies to your research, read the appropriate section before selecting a response.

Materials & experimental systems

n/a	Involved in the study	
[ ]	[X]	Antibodies
[ ]	[X]	Eukaryotic cell lines
[X]	[ ]	Palaeontology and archaeology
[ ]	[X]	Animals and other organisms
[X]	[ ]	Clinical data
[X]	[ ]	Dual use research of concern

Methods

n/a	Involved in the study	
[X]	[ ]	ChIP-seq
[X]	[ ]	Flow cytometry
[X]	[ ]	MRI-based neuroimaging

Antibodies

Antibodies used

EIF4E Cell Signaling Technology 9742
EIF4E Thermo Fisher Scientific MA1-089
ACTB/Actin Cell Signaling Technology 3700
Flag Cell Signaling Technology 8146
EIF4G1 Cell Signaling Technology 2469
EIF4G2 Cell Signaling Technology 2182
Phospho-EIF2S1 (Ser51) Cell Signaling Technology 3398
MKNK1/MNK1 Cell Signaling Technology 2195
Phospho-EIF4E (Ser209) Cell Signaling Technology 9741
MAP1LC3 Cell Signaling Technology 12741
AIFM1/AIF Cell Signaling Technology 5318
Histone H3 Cell Signaling Technology 9715
SOD1 Abcam ab168314HNE Abcam ab46545
ALDH1B1 Proteintech 15560-1-AP
ALDH3A1 Abcam ab129022
ALDH3A2 Thermo Fisher Scientific 15090-1-AP
NOX1 NOVUS NBP1-31546
CPNE3 Thermo Fisher Scientific PA5-56186
PTGES2 Santa Cruz Biotechnology sc-514224
IARS Abcam ab151557
GSTO1 NOVUS NBP1-33763
HSD17B4 Abcam ab97971
CEPT1 Santa Cruz Biotechnology sc-133421
VDAC Cell Signaling Technology 4866
Cytochrome C Cell Signaling Technology 4280
LAMP1 Cell Signaling Technology 9091
β-tubulin Cell Signaling Technology 2128
anti-puromycin Sigma-Aldrich, MABE343, clone 12D10

Validation

Below are validation statements from manufacturers as well as validation performed in-house and by other investigators:
EIF4E Cell Signaling Technology 9742, WB; https://www.cellsignal.com/products/primary-antibodies/eif4e-antibody/9742
EIF4E Thermo Fisher Scientific MA1-089, IP/IF; https://www.thermofisher.cn/cn/zh/antibody/product/eIF4E-Antibody-clone-5D11-Monoclonal/MA1-089?adobe_mc=MCMID%7C29343367619830648330963629541008798385%7CMCAID%3D2F24FEF28515FAD7-40000AA504046E30%7CMCORGID%3D5B135A0C5370E6B40A490D44@AdobeOrg%7CTS%3D1614293705
ACTB/Actin Cell Signaling Technology 3700, WB; https://www.cellsignal.com/products/primary-antibodies/b-actin-8h10d10-mouse-mab/3700
Flag Cell Signaling Technology 8146, WB; https://www.cellsignal.com/products/primary-antibodies/dykddddk-tag-9a3-mouse-mab-binds-to-same-epitope-as-sigma-s-anti-flag-m2-antibody/8146
EIF4G1 Cell Signaling Technology 2469, WB; https://www.cellsignal.com/products/primary-antibodies/eif4g-c45a4-rabbit-mab/2469
EIF4G2 Cell Signaling Technology 2182, WB; https://www.cellsignal.com/products/primary-antibodies/eif4g2-p97-antibody/2182?fromPage=plp&productId=2300
Phospho-EIF2S1 (Ser51) Cell Signaling Technology 3398, WB; https://www.cellsignal.com/products/primary-antibodies/phospho-eif2a-ser51-d9g8-xp-rabbit-mab/3398
MKNK1/MNK1 Cell Signaling Technology 2195, WB; https://www.cellsignal.com/products/primary-antibodies/mnk1-c4c1-rabbit-mab/2195
Phospho-EIF4E (Ser209) Cell Signaling Technology 9741, WB; https://www.cellsignal.com/products/primary-antibodies/phospho-eif4e-ser209-antibody/9741
MAP1LC3 Cell Signaling Technology 12741, WB; https://www.cellsignal.com/products/primary-antibodies/lc3a-b-d3u4c-xp-rabbit-mab/12741
AIFM1/AIF Cell Signaling Technology 5318, WB; https://www.cellsignal.com/products/primary-antibodies/aif-d39d2-xp-rabbit-mab/5318
Histone H3 Cell Signaling Technology 9715, WB; https://www.cellsignal.com/products/primary-antibodies/histone-h3-antibody/9715
SOD1 Abcam ab16831, WB; https://www.abcam.com/superoxide-dismutase-1-antibody-ab16831.html
ALDH1B1 Proteintech 15560-1-AP, WB/IF; https://www.ptglab.com/products/ALDH1B1-Antibody-15560-1-AP.htm
ALDH3A1 Abcam ab129022, WB; https://www.abcam.com/aldh3a1-antibody-epr7406-ab129022.html
ALDH3A2 Thermo Fisher Scientific 15090-1-AP, WB; https://www.thermofisher.cn/cn/zh/antibody/product/ALDH3A2-Antibody-Polyclonal/15090-1-AP?adobe_mc=MCMID%7C29343367619830648330963629541008798385%7CMCAID%3D2F24FEF28515FAD7-40000AA504046E30%7CMCORGID%3D5B135A0C5370E6B40A490D44%40AdobeOrg%7CTS=1614293705
NOX1 NOVUS NBP1-31546, WB; https://www.novusbio.com/products/nox1-antibody_nbp1-31546
CPNE3 Thermo Fisher Scientific PA5-56186, WB; https://www.thermofisher.cn/cn/zh/antibody/product/CPNE3-Antibody-Polyclonal/PA5-56186?adobe_mc=MCMID%7C29343367619830648330963629541008798385%7CMCAID%3D2F24FEF28515FAD7-40000AA504046E30%7CMCORGID%3D5B135A0C5370E6B40A490D44%40AdobeOrg%7CTS=1614293705
PTGES2 Santa Cruz Biotechnology sc-514224, WB; https://www.scbt.com/p/pge-synthase-2-antibody-a-2
IARS Abcam ab151557, WB; https://www.abcam.com/isoleucyl-trna-synthetase-antibody-epr10266b-ab151557.html
GSTO1 NOVUS NBP1-33763, WB; https://www.novusbio.com/products/gsto1-antibody_nbp1-33763
HSD17B4 Abcam ab97971, WB; https://www.abcam.com/hsd17b4-antibody-ab97971.html
CEPT1 Santa Cruz Biotechnology sc-133421, WB; chrome-extension://efaidnbmnnnibpcajpcglclefindmkaj/viewer.html?pdfurl=https%3A%2F%2Fdatasheets.scbt.com%2Fsc-133421.pdf&clen=250497&chunk=true
VDAC Cell Signaling Technology 4866, WB; https://www.cellsignal.com/products/primary-antibodies/vdac-antibody/4866
Cytochrome C Cell Signaling Technology 4280, WB; https://www.cellsignal.com/products/primary-antibodies/cytochrome-c-136f3-rabbit-mab/4280
LAMP1 Cell Signaling Technology 9091, WB; https://www.cellsignal.com/products/primary-antibodies/lamp1-d2d11-xp-rabbit-

mab/9091
β-tubulin Cell Signaling Technology 2128, WB https://www.cellsignal.com/products/primary-antibodies/b-tubulin-9f3-rabbit-mab/2128
4HNE Abcam ab46545 for IF were validated by us and other publication (PMID: 31899616).
anti-puromycin Sigma-Aldrich, MABE343, WB https://www.sigmaaldrich.cn/CN/zh/product/mm/mabe343

Eukaryotic cell lines

Policy information about cell lines and Sex and Gender in Research

Cell line source(s)	HT-1080 ATCC CCL-121<br>Calu-1 ATCC HTB-54<br>PANC1 ATCC CRL-1469<br>HepG2 ATCC HB-8065<br>MEF ATCC SCRC-1008<br>Gpx4-/- Pfa1 Marcus Conrad (Friedmann Angeli et al., 2014)<br>293FT Thermo Fisher Scientific R70007
Authentication	HT-1080, Calu-1, PANC1, HepG2, MEF, and 293FT were authenticated by STR profiling.Gpx4-/- Pfa1 were authenticated by Marcus Conrad.
Mycoplasma contamination	All cell lines used were confirmed to be mycoplasma negative.
Commonly misidentified lines (See ICLAC register)	None of the cell lines used are commonly misidentified lines.

Animals and other research organisms

Policy information about studies involving animals; ARRIVE guidelines recommended for reporting animal research, and Sex and Gender in Research

Laboratory animals	Xenograft studies: 6- to 8-week-old athymic nude female BALB/c mice (Charles River 490)
Wild animals	No wild animals were used in the study.
Reporting on sex	female BALB/c mice
Field-collected samples	No field-collected samples were used in the study.
Ethics oversight	We conducted all animal care and experiments in accordance with the Association for Assessment and Accreditation of Laboratory Animal Care guidelines and with approval from our institutional animal care and use committees (Guangzhou Medical University and UT Southwestern Medical Center).

Note that full information on the approval of the study protocol must also be provided in the manuscript.
